# Juggling skill acquisition through air-toss and ground-bounce practice in college students: A study protocol for the TuJuggling cluster-randomised trial

**DOI:** 10.1371/journal.pone.0361254

**Published:** 2026-11-02

**Authors:** Javier Fernández-Ortega, Andrés B. Fernández-Revelles

**Affiliations:** 1 Department of Physical Education and Sports, Faculty of Sport Sciences, Sport and Health University Research Institute (iMUDS), University of Granada, Granada, Spain; 2 Department of Physical and Sports Education, Faculty of Sport Sciences, University of Granada, Granada, Spain; Shahid Chamran University of Ahvaz, IRAN, ISLAMIC REPUBLIC OF

## Abstract

**Introduction:**

Practice organization is vital for motor acquisition, yet pragmatic evidence on how instructional constraints shape learning in authentic settings remains scarce. The TuJuggling protocol establishes a standardised assessment and training framework for complex bimanual skills in novices using an ecological dynamics perspective.

**Methods:**

In this cluster-randomised crossover trial, registered on ClinicalTrials.gov (Identifier: NCT07528924), 200 students will complete two 4-week juggling interventions: air-toss (A) and floor-rebound (B), separated by a 1-week washout. The primary outcome is a Global Composite Score (40 tasks) across stable (floor) and unstable (BOSU) surfaces. Secondary outcomes include cognitive functions (attentional/inhibitory control, visuospatial memory, and working memory), handgrip strength, and psychological factors (flow and affect). Assessments occur at baseline and after each intervention period.

**Results:**

Data will be analysed using linear mixed models (LMM) to evaluate the effects of method, period, and sequence while accounting for the cluster-based design. Missing data will be handled via Full Information Maximum Likelihood (FIML). Mediation models will further investigate whether psycho-cognitive shifts explain the observed motor training effects.

**Discussion:**

This protocol provides a rigorous framework to evaluate how physical constraints and exposure order shape coordination. Findings will offer evidence-based recommendations for sequencing motor skill progressions in higher education, bridging theoretical motor learning and applied pedagogy.

## 1 Introduction

The acquisition of complex motor skills is fundamentally shaped by practice organization, encompassing decisions on whether skills are practiced as whole or part tasks and how practice is scheduled over time [1]. In particular, practice order and the alternation between movement patterns can significantly influence not only acquisition but also retention [2] and transfer [3], a phenomenon extensively explored in the contextual interference literature [4,5]. Juggling serves as a particularly useful model for examining these learning trajectories because it requires sustained bimanual coordination and visuomotor control [6], making it sensitive to practice organization and ideal for identifying marked interindividual differences in performance [7]. From an ecological dynamics perspective, skill performance is understood as emerging from the continuous interaction between the learner, the task, and environmental constraints [8], which supports the use of practice designs that promote exploration and adaptive coordination through the manipulation of task-led constraints [8].

Consistent with this theoretical framework, the present study compares two juggling training methods grounded in distinct pedagogical and biomechanical logics: tossing-based juggling and bouncing-based juggling. From a constraints-led perspective, these methods impose different perceptual-motor demands that shape coordination patterns [8]. While tossing-based juggling emphasizes the control of flight time and anticipatory timing under relatively predictable trajectories, it often introduces high vertical variability and spatial uncertainty. In contrast, bouncing-based juggling utilizes the floor as a physical boundary to standardize the apex of the trajectory, thereby reducing uncertainty [9]. Furthermore, the extended duration of the ball’s path from the hand to the floor and back increases the total flight time, providing a larger temporal window for the learner to process sensory feedback and execute motor corrections [10]. This approach aligns with the principle of “task scaling,” where the temporal and spatial constraints are modified to better match the learner’s capabilities [11], potentially leading to more stable coordination patterns [7].

Despite the theoretical promise of task scaling, a considerable part of the evidence on practice-order effects originates from highly controlled paradigms, and the generalizability of these benefits to complex, real-world skills in applied settings has been explicitly questioned [12,13]. In education-oriented contexts, systematic evidence indicates that physical-education-based interventions can produce meaningful learning, but outcomes vary significantly across programs and implementation conditions [14,15]. There is a critical need for pragmatic research embedded in authentic instructional settings [16] that tests concrete approaches under real-world constraints [14]. To address this gap, the current study adopts a two-period, two-sequence cross-over design assigned at the class-group level [17,18]. This design allows for the direct comparison of methods within the same individuals while reducing inter-individual variability [19] and accounting for the fact that motor learning effects typically persist and influence subsequent performance in complex skills [2,7].

Juggling has also been proposed to place some demands on visuospatial processing, sustained attention, and executive control, suggesting its potential as a task for examining interactions between motor and cognitive processes [20]. Evidence indicates that coordinative training may be accompanied by changes in cognitive performance and neural processing [21], although these effects are task-dependent [10]. In older adults, this cognitive engagement provides a rationale for exploring associations between performance improvement and cognitive [22]. Within this context, transfer is operationalised as relative performance across different execution contexts such as stable versus unstable ground [22,23], while performance stability is described as the maintenance of gains across assessment time points [7].

For instance, within the same “Motor Games” course that supplies the recruitment pool, identifying which instructional constraint more reliably supports skill acquisition and transfer has direct pedagogical value, informing how instructors sequence bimanual coordination content for future cohorts of Physical Education trainees.

These cognitive measures were selected to map onto specific demands of the juggling task. Attentional/inhibitory control (Go/No-Go) is hypothesised to relate to real-time suppression of anticipatory errors during ball release: when the timing of a throw or catch is disrupted, the performer must inhibit the planned motor response and reprogram it within milliseconds, a process closely mirrored in fast-ball sports, where experts reduce anticipation-timing costs through stronger inhibition of an incorrect prepared response [23] via inhibitory processing in the right inferior frontal gyrus [24], consistent with the broader link between inhibitory control and sport performance [25]. Visuospatial memory (Matrix Rotation) is hypothesised to index the continuous construction and updating of spatial representations of ball trajectories needed to time throws and catches; mental rotation shares neural substrates with spatial motor transformation and predicts performance in spatially demanding motor tasks, and juggling training itself has been shown to enhance mental rotation performance in children [26]. Working memory (Corsi) is hypothesised to index the visuospatial working-memory load of maintaining and updating the positions of multiple balls in flight; visuospatial working memory predicts both the rate and the chunking organisation of newly acquired motor sequences [27].The primary objective of this study is to compare the effectiveness of these two training methods on overall juggling performance. Secondarily, we aim to describe and compare temporal changes in performance across three assessment points, baseline, Evaluation 1, and Evaluation 2, and estimate differences attributable to exposure sequence (A → B vs B → A). We further explore whether changes in juggling performance are associated with attentional/inhibitory control, visuospatial memory, working memory, handgrip strength, and psychological states of affect and flow. We hypothesize that bouncing-based juggling will result in greater performance gains than tossing-based juggling, particularly in the early stages, due to the increased temporal window for feedback. Consequently, we expect juggling performance and the magnitude of change to differ by exposure sequence, reflecting a learning-order effect. Finally, we anticipate that larger improvements in juggling performance will be positively associated with changes in cognitive performance, affective responses, and the flow state experienced during practice.

## 2 Methods

### 2.1 Study design, registration, and ethics

The study is a two-period, two-sequence cluster-randomised crossover intervention trial (A → B and B–A) with repeated measurements at baseline, Evaluation 1, and Evaluation 2, as illustrated in the study SPIRIT schedule (Fig 1), the complete SPIRIT checklist is provided in S1 File. Sequence allocation is performed at the class-group level to minimize contamination [28]. Participation is entirely voluntary, and all students must provide electronic informed consent via Google Forms prior to any research-related data collection. A clear distinction is maintained between mandatory academic activities and voluntary research participation, ensuring that the decision to participate has no impact on academic standing or grading.

**Fig 1:**
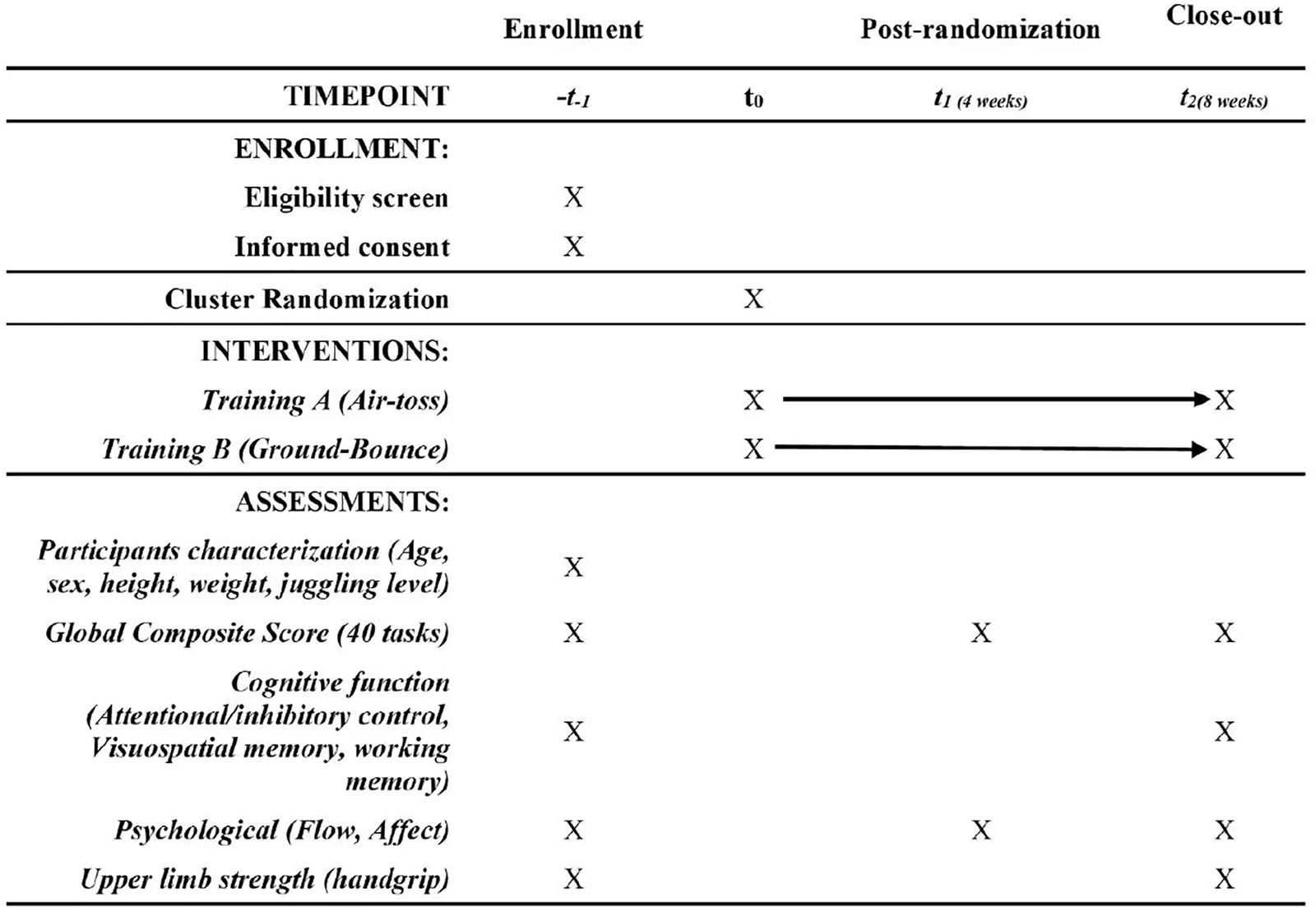
SPIRIT schedule of enrollment, interventions, and assessments.

The TuJuggling trial is designed as a multi-cohort study with participant recruitment and data collection organized in two sequential waves to achieve the required sample size. Recruitment for the first wave (Wave 1) started on 23/03/2026 and was completed in 31/03/2026. Recruitment for the second wave (Wave 2) is scheduled to start in February 2027. Consequently, data collection for the complete trial is anticipated to conclude by June 2027, with primary analysis and results expected by September 2027.

The protocol is reported in accordance with the Standard Protocol Items: Recommendations for Interventional Trials (SPIRIT) 2025 Statement [29] and is registered on ClinicalTrials.gov (Identifier: NCT07528924, submitted on 18 February 2026). The full study protocol is available in S3 File. In accordance with the Declaration of Helsinki, the study was approved by the Ethics Committee on Research of the University of Granada (Ref: 5654/CEIH/2026). This protocol was prepared in accordance with the SPIRIT 2025 statement; the completed checklist (S1 File) details the location of all 34 standard protocol items throughout the manuscript.

### 2.2 Participants and recruitment

The study aims to include a pragmatic sample of 200 students enrolled in the “Motor Games” course at the University of Granada. To reach the target sample size of 200 students while maintaining the pragmatic nature of the study within the ‘Motor Games’ course, recruitment is conducted across two consecutive academic cycles (Wave 1: Spring 2026; Wave 2: Spring 2027). Although this study adopts a pragmatic recruitment approach, the sample size was validated using Monte Carlo simulations based on a Minimum Detectable Difference (MDD) framework [30]. The Minimum Detectable Difference was estimated via a custom Monte Carlo simulation implemented in R (v4.5.3; dplyr, tibble, purrr, tidyr, readr packages), running 1,500 simulated datasets per candidate effect size for each combination of ICC (0.01, 0.03, 0.05) and baseline-Evaluation 1 correlation (0.30, 0.50, 0.70) examined. Under conservative assumptions, including 6 clusters, an intra-class correlation (ICC) of 0.03, a baseline-post correlation of r = 0.50, and a standard deviation (SD) of approximately 22.5 points for the primary composite score (derived from preliminary descriptive baseline data from the first recruitment wave, Wave 1; this estimate is based on a single wave and time point and should be interpreted as provisional), a sample of 200 participants provides approximately 80% power to detect method differences of 10–11 points (approximately 6% of the 0–192 theoretical range of the primary outcome; see Sect 2.4), consistent across a grid of ICC (0.01–0.05) and correlation (0.30–0.70) assumptions (range: 8.98–16.02 points). To detect a minimally relevant difference of 12 points (representing ~2 points per task), a minimum of 155 participants with complete data is required. Accounting for an estimated 10% dropout rate, we have targeted a minimum recruitment of 173 students. This ensures the study is sufficiently powered to address the primary objective during Period 1, even before considering the additional statistical power inherent in the repeated-measures structure of the full crossover design.

The inclusion criteria are participants must be at least 18 years old and enrolled in the course. We exclude students with injuries or visual impairments that prevent safe practice, as well as those with previous high-level juggling proficiency (>20 catches of a 3-ball cascade at baseline) to avoid ceiling effects [7]. Allocation of the six class-groups to sequences is performed via a two-stage physical randomization procedure conducted by an individual external to the research team. In the first stage, the six available class-groups are identified and assigned a numerical ID. In the second stage, a dice-throwing method was employed to randomly allocate these clusters [31] into two arms: three groups are assigned to Sequence 1 (Method A → Method B) and the remaining three to Sequence 2 (Method B → Method A). This procedure ensures that the assignment of the training method order is entirely random and independent of the researchers’ influence, maintaining allocation concealment until the baseline assessment is completed [32].

### 2.3 Intervention and adherence monitoring

The program lasts 12 weeks, with two four-week training periods separated by a one-week washout interval. During this washout week, participants are instructed to maintain their normal daily routines but are strictly prohibited from engaging in any form of juggling practice to minimize carry-over effects. Participants are expected to complete 5 sessions per week a total of 20 per period. Practice is self-directed and supported by standardised video tutorials (detailed in S2 File) where students advance to the next pattern upon achieving 20 consecutive successful catches, an advancement criterion applied identically to both the air-toss and ground-bounce interventions, which are otherwise matched in volume and session structure and differ only in task constraints. S2 File comprises 14 video tutorials in total (8 for the air-toss progression and 6 for the ground-bounce progression). Each of the 14 patterns is broken down in the video tutorials into a graded sequence of sub-steps that generally follows the same instructional logic: unimanual practice with the dominant hand, then the non-dominant hand, then hand-to-hand alternation, then bimanual coordination with an increasing ball count, and finally continuous execution, the same criterion used for the 20-consecutive-catch advancement rule. Attendance and compliance are monitored via an online spreadsheet where students record date, minutes, and tasks performed. To ensure analytical precision, these metrics are defined as follows: (i) Attendance is defined as a session in which the date and minutes are recorded, regardless of duration; (ii) Compliance refers to an attended session with a recorded duration of ≥20 minutes; and (iii) Adherence, the primary metric of program exposure, is calculated as the percentage of planned sessions that are both attended and compliant.

The primary analysis follows an intention-to-treat (ITT) principle, with missing outcome data handled via Full Information Maximum Likelihood as detailed in Sect 2.8, but a per-protocol (PP) (28) analysis will be conducted for participants achieving at least 60% adherence (≥12 compliant sessions per period), a dosage likely to induce stable changes in bimanual coordination [33].

### 2.4 Juggling assessment and primary outcome

The primary outcome is a Global Composite Score (GCS) derived from a battery of 40 tasks. This battery is composed of two identical sets of 20 tasks: one performed on a stable surface (floor) and one on an unstable surface (BOSU). As detailed in Table 1, each set includes basic air-toss patterns (7 tasks), basic ground-bounce patterns (7 tasks), and training-specific progression patterns (6 tasks). These tasks follow a hierarchical complexity, ranging from single-ball throws to 3-ball cascade variations. All assessment procedures are visually supported by the video tutorials available in S2 File.

**Table 1:** Composition of the Juggling Assessment Battery (20 tasks per surface condition).

Block	Uni-manual Tasks (1H)	Bi-manual Tasks (2H)	Description
Air-Toss (7)	1B (R/L), 2B Cycle (R/L)	2B Columns, 2B Alternating, 3B Cascade	Parabolic air throws
Bounce (7)	1B (R/L), 2B Cycle (R/L)	2B Columns, 2B Alternating, 3B Bounce	Floor-rebound patterns
Training-Specific (6)	—	A, C, CH, RA, RB, RC	Advanced patterns
Total per Surface	8 tasks (Max 24 pts)	12 tasks (Max 72 pts)	20 tasks total

*Note:* 1H/2H = 1 or 2 hand; 1B/2B/3B = 1, 2, or 3 balls; R/L = Right and Left hand; A = Standard Cascade; C = Cascade with Cycle; CH = Successive Columns; RA/RB/RC = Rebound Active/Passive/Imperative. Scoring: 0.1 pts per catch (1H max = 3.0 pts; 2H max = 6.0 pts).

To prioritize the absolute volume of motor success and the coordinative demands of bimanual tasks, a Fixed Rate Scoring system was implemented, following the methodological principles of previous juggling research where total catch counts serve as the primary proxy for proficiency [34]. The individual Task Score is calculated by multiplying the number of actual catches achieved by a constant value of 0.1 points. This ensures that every successful catch maintains an identical weight across all experimental conditions. The Global Composite Score has a theoretical range of 0–192 points (24 points across 8 uni-manual tasks plus 72 points across 12 bi-manual tasks, per surface, summed across both surfaces). The higher maximum attainable for bimanual tasks is a deliberate design choice: because bimanual tasks are expected to show the greatest inter-individual variance in motor learning, this scoring structure ensures they contribute proportionally more to the total score, increasing the composite’s sensitivity to the intervention’s impact. Scoring reliability will be assessed via intra-rater re-scoring of a random 10% subsample of videos to detect systematic errors and document scoring consistency.

The stopping criteria are set at 30 catches for one-hand tasks (yielding a maximum of 3 points) and 60 catches for two-hand tasks (yielding a maximum of 6 points) to prevent muscle fatigue [6]. This scoring logic ensures that high-volume bimanual tasks, where the greatest learning variance is expected, contribute more to the final score than simpler uni-manual tasks, thus avoiding “ceiling effects” from masking the intervention’s impact. The GCS is calculated as the sum of these absolute values across all 40 tasks. To ensure robust results, scoring is conducted by a blinded evaluator (AF-R) using anonymised video recordings and standardised criteria [35].

### 2.5 Secondary measure

Secondary variables include psychological affect (PANAS; full 20-item version, 10 items per subscale) (32) and flow states [36]. Cognitive function variables, attentional/inhibitory control (Go/No-Go test), visuospatial memory (Matrix Rotation test), and working memory (Corsi test), are evaluated via the PEBL platform [37]. Additionally, handgrip strength is assessed using digital dynamometry (TKK 5101 Grip D, Takey, Tokyo, Japan) to explore physical predictors of motor learning. The procedure is as follows standing position, arm alongside the body, unsupported; grip span standardised at 5.5 cm for males and 4.5 cm for females; two maximal attempts per hand, alternating hands, highest value retained.

### 2.6 Descriptive variables and anthropometrics

At baseline, participants’ demographic and anthropometric data will be collected, including age (years), sex given at birth (Male or Female), height (m), and weight (kg). Body Mass Index (BMI) will be calculated as weight in kilograms divided by the square of height in meters (kg/m^2^). Additionally, manual laterality (handedness) will be assessed via self-report to identify dominant hand use (right-handed or left-handed). A summary of all primary, secondary, and descriptive variables, along with their respective assessment tools, is provided in Table 2.

**Table 2:** Summary of primary and secondary outcome measures, assessment tools, and timing.

Category	Variable	Assessment Tool/Method
Demographic	**Age**	Self-report questionnaire
	**Sex**	Self-report questionnaire
	**Manual Laterality**	Self-report questionnaire
Anthropometrics	**Height,**	Stadiometer
	**Weight**	Digital scales
Primary Outcome	**Juggling Performance**	Composite score of 40 tasks (20 Stable and 20 BOSU)
Secondary	**Cognitive function**	Attentional/Inhibitory control, Visuospatial memory and Working memory
	**Handgrip Strength**	Manual Dynamometry (kg)
	**Flow & Affect**	Flow State Scale & PANAS (Positive/Negative Affect)
Adherence	**Attendance and Compliance**	Digital logs (20 sessions of 20 minutes)

### 2.7 Data management, blinding, safety and dissemination

Due to the pragmatic nature of this trial conducted within a university Physical Education course, participants and instructors cannot be blinded to the intervention groups. However, several measures have been implemented to minimize detection and analysis bias. First, the assessment of the primary juggling outcome is performed through video recordings. A blinded outcome assessor will score the 40-task battery according to standardized rubrics. To ensure concealment during the scoring process, a double-layer anonymization system is employed: each participant is identified only by a numeric study ID and a unique “avatar” (e.g., playing card/object combinations, such as ‘Q-Diamond-Fire’ (240 possible unique pairings)) visible in the recording. This prevents the assessor from linking the juggling performance to specific individuals or their allocated intervention sequence. Additionally, data analysts will remain blinded to group allocation; videos are relabelled with the study ID and assessment time point only (no group, sequence, or period information) by a data manager independent of the outcome assessor, who has no access to the main research database during the primary statistical processing.

Safety is prioritised through an adverse event monitoring protocol; participants are instructed to stop activity if they experience pain, dizziness, or significant discomfort, following the official Accident Response Protocol of the Faculty of Sport Sciences.

To ensure the effective communication of the trial results to the participants, an individual feedback report will be provided to each student upon completion of the study. This report will detail the participant’s performance in cognitive tasks, physical strength, and juggling skills, with results presented in percentiles relative to the study population to facilitate self-evaluation. Furthermore, once the final results of the TuJuggling trial are published in a peer-reviewed scientific journal, a direct link to the publication will be sent to all participants. The study findings will also be updated on the ClinicalTrials.gov registry (NCT07528924) and disseminated through institutional communication channels at the University of Granada. Upon study completion, a de-identified individual-level dataset and the associated R analysis code will be made available via the Open Science Framework (OSF; https://doi.org/10.17605/OSF.IO/BKCGQ), subject to a data use agreement to protect participant confidentiality.

### 2.8 Statistical analysis plan

Analyses will be conducted in R (version 4.5.3 or higher) using linear mixed models (LMM). Statistical significance will be defined at an alpha level of 0.05, and all tests will be two-tailed. To address the small number of clusters (k = 6) and prevent Type I error inflation, the Period 1 comparison will follow a two-stage covariate-adjusted cluster-level analysis [18]. Longitudinal models will include fixed effects for Method, Period, and Sequence, with random intercepts for participants [38]. Assumptions for LMM (normality, homoscedasticity, and linearity) will be verified through residual analysis, and any potential outlying data will be assessed using Cook’s distance. For the secondary longitudinal model, Satterthwaite degrees of freedom [39], as implemented natively in the LMMstar package [40], will be used for fixed-effect inference. To formally examine the possibility of a treatment-by-period interaction (equivalently, differential carryover), we additionally apply Grizzle’s [41] classical two-stage diagnostic approach, adapted to the cluster-level framework used for the primary analysis: for each participant, the sum of Evaluation 1 and Evaluation 2 GCS is computed; these subject-level sums are averaged by cluster and compared between the two sequence groups (A → B vs. B → A) using an independent t-test with k-2 = 4 degrees of freedom, at the conventional diagnostic significance threshold (α = 0.10). A significant result would provide additional support for anchoring the primary treatment effect estimate to the Period 1 comparison. Mediation models (5,000 bootstrap iterations) will investigate whether psycho-cognitive shifts explain the training effects. Five candidate mediators are pre-specified: flow state, affect (PANAS), attentional/inhibitory control (Go/No-Go), working memory (Corsi), and visuospatial memory (Matrix Rotation). For each, Path A (effect of Method on change in the mediator) and Path B (association between mediator change and post-intervention juggling performance, adjusting for baseline) will be estimated using the PROCESS macro framework (bruceR implementation in R). Missing data will be handled using Full Information Maximum Likelihood (FIML), assuming data are missing at random. Beyond P-values, results will include 95% confidence intervals and effect sizes to provide a measure of precision and magnitude for all primary outcomes. To account for the number of secondary outcome tests conducted, a Benjamini-Hochberg false discovery rate correction will be applied across the family of secondary outcomes (PANAS, flow, the three cognitive measures, and handgrip strength); the primary GCS analysis is treated as confirmatory and is not subject to this correction.

## 3 Discussion

This protocol describes a pragmatic trial designed to determine whether the physical constraints of the learning method (Air-toss vs. Ground-bouncing) and the order of exposure (A → B vs. B → A) significantly shape the acquisition of complex motor skills. Juggling serves as an ideal model for this investigation because coordinative skills often exhibit non-linear learning patterns that traditional “pre-post” designs fail to capture [7]. By utilising a high-frequency longitudinal design (Baseline, Evaluation 1, Evaluation 2), we expect to identify critical convergence or divergence points between instructional methods. Theoretically, we anticipate that starting with ground-bounces may provide a “scaffolding” effect, reducing temporal pressure and allowing for a more stable initial coordination pattern, which could then facilitate the transition to more complex air-tosses through positive transfer [2,4].

Although direct comparisons between air-toss and ground-bouncing are scarce in current literature, previous studies on juggling have consistently shown that the acquisition of this complex skill is highly sensitive to practice organization [42]. For instance, research on contextual interference suggests that while high-interference schedules (e.g., alternating tasks) might slow down initial performance, they often lead to superior long-term retention and transfer compared to repetitive practice [3,4]. By comparing two distinct physical constraints (Air vs. Floor), this trial extends this debate to the very nature of the task’s entry point, exploring whether “simpler” temporal constraints (bouncing) act as a form of implicit learning scaffold.

Furthermore, the existing literature highlights a strong but complex link between motor mastery and cognitive domains [43,44]. Previous neuroimaging and behavioral studies suggest that juggling practice may be associated with structural brain plasticity [44–46], reflecting structural (voxel-based morphometry) and functional/temporal (EEG-based) evidence, respectively, and improvements in working memory [33]. However, there is a “gap” regarding whether specific methods (such as the Ground-bouncing method) might reduce the initial cognitive load compared to traditional methods. By integrating the cognitive function tests, this protocol will discuss whether initial cognitive profiles, specifically attentional/inhibitory control, visuospatial memory and working memory, predict the rate of success across the two different training modalities, a relationship that remains under-explored in pragmatic educational settings.

A notable challenge in juggling research is the lack of standardised assessment tools, particularly for novice populations. While previous literature has frequently focused on expert performance [6] or utilised restricted metrics (e.g., only the 3-ball cascade) [47], there is a significant gap in evaluating the early stages of skill acquisition where progress is often non-linear and fragmented. The development of the extensive 40-task assessment battery in the TuJuggling protocol specifically addresses this limitation. By evaluating a wide spectrum of patterns, ranging from simple one-ball manipulations to training-specific complex cycles, this battery is designed to capture the granular nuances of beginner progress. This approach not only allows us to identify the ‘learning ceiling’ for novices but also provides the necessary sensitivity to discriminate between participants who achieve high-level bimanual mastery and those who remain at fundamental coordination levels, a distinction that simpler scales often fail to detect.

The primary strength of this study is its high ecological validity, achieved through standardised instructional content within a naturalistic university setting. This ecological strength is complemented by the internal-validity features of the design: prospective registration, cluster-randomised allocation, blinded video-based outcome assessment, and a purpose-built 40-task battery spanning the full range of novice-to-advanced juggling performance. Several limitations should also be acknowledged. First, the pragmatic, classroom-embedded design precludes blinding participants and instructors to group allocation, risking expectancy or Hawthorne effects on self-directed practice; this is partly mitigated by fully blinded, catch-count-based outcome assessment. Second, the one-week washout is not expected to erase the general juggling knowledge gained in Period 1, since comparable motor skills are typically retained over months, not days [48]. Protection instead rests on counterbalanced sequence allocation and the pre-specified carryover diagnostic in Sect 2.8, which anchors the primary effect to the Period 1 comparison if needed; the one-week interval reflects the academic calendar rather than an assumption that it eliminates carryover. Third, cross-group contamination was addressed through explicit practice logs and separate distribution of materials. Fourth, self-reported adherence logs are susceptible to recall and social desirability bias in unsupervised, student-led practice, and no independent verification is implemented.

If significant differences are found, the educational implications are substantial: findings could redefine how juggling and other bimanual skills are sequenced in Physical Education curricula. Specifically, an early emphasis on modified constraints (e.g., bouncing) might yield more robust performance under altered conditions, such as the postural demands of the BOSU balance trainer.

We intend to incorporate a delayed retention assessment, although its feasibility within the constraints of the academic calendar has not yet been confirmed and any such assessment is expected to be affected by a substantially lower participation rate than the main trial. Absent that, future studies should incorporate delayed retention tests, ideally several months after course completion, to distinguish transient gains from permanent learning stability. Additionally, expanding the cognitive battery to include neurobiological measures (e.g., fNIRS) would provide deeper insight into the psycho-cognitive shifts that accompany motor mastery in educational environments.

In conclusion, the TuJuggling protocol addresses the critical need for methodological standardization in the study of juggling, a versatile and complex motor skill. By introducing a comprehensive 40-task assessment battery specifically tailored for novice populations, this study establishes a robust foundation for evaluating motor mastery beyond traditional, oversimplified metrics. Ultimately, this framework serves as a solid knowledge base to refine training and evaluation strategies in Physical Education. By identifying the most effective instructional constraints (Air-toss vs. Ground-bounce), we expect not only to optimize the learning process for students but also to provide a replicable model that ensures more consistent and high-quality results in future motor learning research.

## Supporting information

S1 File SPIRIT 2025 Checklist. Completed SPIRIT checklist detailing the location of protocol items within the manuscript.
(DOCX)

S2 File Juggling Training Resources. Structured list and hyperlinks to the 14 standardized video tutorials for air-toss and ground-bounce training progressions.
(DOCX)

S3 File Full Study Protocol. Complete study protocol of the TuJuggling cluster-randomised trial.
(DOCX)
