# Supplementary material for: Juggling skill acquisition through air-toss and ground-bounce practice in college students: A study protocol for the TuJuggling cluster-randomised trial
Source: PLoS One. 2026 Nov 2;21(11):e0361254. doi: 10.1371/journal.pone.0361254 (PMC13726955; doi:10.1371/journal.pone.0361254)
Supplement: Supplementary file 2 [file pone.0361254.s002.docx]

**S2 File. Juggling Training Resources**

The S2 File provides a set of 14 standardized video tutorials, hosted on an open-access platform (*Google Sites - University of Granada*), designed to guide the acquisition of complex bimanual coordination skills. The resources are structured according to the two intervention arms of the crossover trial:

***Intervention A (Air-Toss Juggling)***

- **Standard cascade**: <https://sites.google.com/go.ugr.es/malabares/lanzamiento-progresiones/a-cascada-normal?authuser=0>
- **Cascade – cycle progression**: <https://sites.google.com/go.ugr.es/malabares/lanzamiento-progresiones/b-cascada-progresi%C3%B3n-ciclos?authuser=0>
- **Cascade and 423 cycle**: <https://sites.google.com/go.ugr.es/malabares/lanzamiento-progresiones/c-cascada-y-ciclo-423?authuser=0>
- **Successive columns**: <https://sites.google.com/go.ugr.es/malabares/lanzamiento-progresiones/ch-columnas-sucesivas?authuser=0>
- **Columns**: <https://sites.google.com/go.ugr.es/malabares/lanzamiento-progresiones/d-columnas?authuser=0>
- **Shower or SAR wheel**: <https://sites.google.com/go.ugr.es/malabares/lanzamiento-progresiones/e-ducha-o-rueda-sar?authuser=0>
- **Shower or SCAR wheel**: <https://sites.google.com/go.ugr.es/malabares/lanzamiento-progresiones/f-ducha-o-rueda-scar?authuser=0>
- **Over-the-head juggling (3 balls)**: <https://sites.google.com/go.ugr.es/malabares/lanzamiento-progresiones/g-por-encima-de-la-cabeza-3b?authuser=0>

***Intervention B (Ground-Bounce Juggling):***

- **RA progression: Active bounce**: <https://sites.google.com/go.ugr.es/malabares/rebote-progresiones/progresi%C3%B3n-ra-rebote-activo?authuser=0>
- **RB progression: Passive bounce**: <https://sites.google.com/go.ugr.es/malabares/rebote-progresiones/progresi%C3%B3n-rb-rebote-pasivo?authuser=0>
- **RC progression: Imperative bounce**: <https://sites.google.com/go.ugr.es/malabares/rebote-progresiones/progresi%C3%B3n-rc-rebote-imperativo?authuser=0>
- **RD progression: Active bounce (supination)**: <https://sites.google.com/go.ugr.es/malabares/rebote-progresiones/progresi%C3%B3n-rd-rebote-activo-supinaci%C3%B3n?authuser=0>
- **RE progression: Table bounce**: <https://sites.google.com/go.ugr.es/malabares/rebote-progresiones/progresion-re-encima-mesa?authuser=0>
- **RF progression: Wall bounce (3 balls)**: <https://sites.google.com/go.ugr.es/malabares/rebote-progresiones/progresi%C3%B3n-rf-rebote-en-pared-3b?authuser=0>
