# Supplementary material for: Juggling skill acquisition through air-toss and ground-bounce practice in college students: A study protocol for the TuJuggling cluster-randomised trial
Source: PLoS One. 2026 Nov 2;21(11):e0361254. doi: 10.1371/journal.pone.0361254 (PMC13726955; doi:10.1371/journal.pone.0361254)
Supplement: Supplementary file 3 [file pone.0361254.s003.docx]

TuJuggling: a cluster-randomized cross-over trial comparing two juggling learning methods in college students

Javier, Fernández-Ortega^1^ and Andrés B., Fernández-Revelles^2^

^1^ PROFITH "PROmoting FITness and Health Through Physical Activity" Research Group, Sport and Health University Research Institute (iMUDS), Department of Physical and Sports Education,Faculty of Sport Sciences, University of Granada, Granada, Spain

^2^ Department of Physical and Sports Education,Faculty of Sport Sciences, University of Granada, Granada, Spain

[Clinicaltrials.gov; Identifier: NCT07528924](https://clinicaltrials.gov/study/NCT07528924?cond=juggling&aggFilters=status:not%20rec&viewType=Card&rank=1)

April 17^th^ 2026

# 1. Introduction

The learning of complex motor skills is shaped by how practice is organized, including decisions about whether skills are practiced as whole vs. part tasks and how practice is scheduled over time (Brydges et al., 2007). In particular, practice order and alternation between movement patterns (e.g., blocked vs. random schedules) can influence not only acquisition but also retention (Czyż, Wójcik, Solarská, et al., 2024) and transfer (Czyż, Wójcik, & Solarská, 2024), consistent with the contextual interference literature and its extensions to more complex skills and longer training schedules (Porter & Magill, 2010; Wu et al., 2011). However, a considerable part of the evidence on practice-order effects originates from highly controlled paradigms, and the generalizability of contextual interference benefits to complex, real-world skills has been explicitly questioned, motivating studies in applied settings (Magelssen et al., 2022; Pauwels et al., 2014).

In education-oriented contexts, systematic evidence indicates that physical-education-based interventions can produce meaningful learning and development effects (Dudley et al., 2022), but outcomes vary across programs and implementation conditions (Lorås, 2020), reinforcing the need for pragmatic research embedded in authentic instructional settings (Telford et al., 2021). Accordingly, when cluster allocation is used to reduce contamination in class-based delivery (Keogh-Brown et al., 2007), reporting and design decisions should follow cluster-trial guidance (Campbell et al., 2012) and standard protocol items (A.-W. Chan et al., 2025) to support transparent interpretation and reproducibility.

Juggling is a complex motor skill that requires sustained bimanual coordination and visuomotor control (Gerber et al., 2014), making it a useful model for examining learning trajectories and marked interindividual differences in performance (Geller et al., 2023). From an ecological dynamics perspective, skill performance is understood as emerging from the continuous interaction between the learner and task and environmental constraints (Renshaw & Chow, 2019), which supports the use of practice designs that promote exploration and adaptive coordination, particularly in educational and sport contexts (Renshaw & Chow, 2019). Consistent with this view, juggling learning patterns have been shown to differ depending on how practice is structured (e.g., progressively increasing difficulty vs. learning the complete skill), indicating that this type of task is sensitive to practice organization and can yield distinct learning trajectories (Geller et al., 2023). Evidence from juggling further indicates that how the skill is partitioned and practiced (whole vs. part) can meaningfully influence learning outcomes, reinforcing the relevance of practice organization in this domain (J. S. Y. Chan et al., 2015).

From a pedagogical standpoint, embedding the intervention within regular course delivery provides an authentic context to evaluate instructional strategies without disrupting academic routines, which aligns with the logic of pragmatic education-based trials (Telford et al., 2021) and the integration of undergraduates in research processes (Rogers et al., 2012). In physical education more broadly, systematic evidence indicates that curriculum-based programs can improve motor competence, although effects vary across outcomes and implementation conditions (Dudley et al., 2022), supporting the need to test concrete approaches under real-world constraints (Dudley et al., 2022). Consistent with these observations, pragmatic cluster-based school interventions also highlight variability in implementation and outcomes, underscoring the importance of robust design, transparent reporting (Hoffmann et al., 2014), and appropriate handling of clustering when instruction is delivered at the class level (Telford et al., 2021).

Within this applied framework, the present study compares two juggling training methods grounded in distinct pedagogical and biomechanical logics: (A) throwing-based juggling and (B) bouncing-based juggling (rebounds from the floor). From a constraints-led perspective, these methods impose different perceptual-motor demands that shape coordination patterns (Renshaw & Chow, 2019). Throwing-based juggling emphasizes the control of flight time and anticipatory timing under relatively predictable trajectories, yet it introduces high vertical variability, as inconsistent toss heights can increase uncertainty and task complexity.

In contrast, bouncing-based juggling introduces a ball–surface interaction that may facilitate early skill acquisition through two primary mechanisms. First, the floor acts as a physical boundary that standardizes the apex of the trajectory, ensuring a consistent travel distance and reducing the spatial uncertainty inherent in air-tosses. Second, the extended duration of the ball’s path, from the hand to the floor and back, increases the total flight time, providing a larger temporal window for the learner to process sensory feedback and execute motor corrections (Malik et al., 2022). This approach aligns with the principle of "task scaling," where the temporal and spatial constraints of the task are modified to better match the learner's capabilities (Iwatsuki & Regis, 2021), potentially leading to more stable coordination patterns during the initial stages of learning. By manipulating these task constraints, it may leads to distinct learning trajectories and adaptive solutions rather than uniform performance gains, particularly in complex coordinative tasks (Geller et al., 2023).

To address these questions, the present study adopts a two-period, two-sequence cross-over design (A–B and B–A) assigned at the class-group level (Rietbergen & Moerbeek, 2011; Wellek & Blettner, 2012). This design allows direct comparison of training methods while reducing inter-individual variability (Hui et al., 2015) and limiting contamination within educational groups. Importantly, motor learning is not expected to dissipate between practice periods; rather, learning effects typically persist and influence subsequent performance, particularly in complex skills practiced over multiple sessions (Czyż, Wójcik, Solarská, et al., 2024; Geller et al., 2023). Accordingly, estimation of the method effect is prioritized using Period 1, where prior exposure to the alternative method cannot occur, whereas Period 2 is interpreted as complementary information reflecting order, transfer, and potential carry-over effects (Kulnik et al., 2025; Shi & Ye, 2024). Within this framework, transfer is operationalized as relative performance across different execution contexts stable ground vs. unstable ground (Laudner & Koschnitzky, 2010; Moreno et al., 2023), while performance stability is described as the maintenance of performance across assessment time points, consistent with contemporary approaches to studying learning dynamics rather than isolated endpoint outcomes (Geller et al., 2023).

Finally, juggling has been consistently associated with substantial demands on visuospatial processing, sustained attention, and executive control, supporting its use as a paradigmatic task for examining interactions between motor and cognitive processes (Voelcker-Rehage et al., 2011). Longitudinal and experimental evidence indicates that coordinative training, including juggling-based interventions, may be accompanied by changes in cognitive performance and neural processing (Latino et al., 2021), although effects vary by task, population, and training characteristics (Malik et al., 2022; Voelcker-Rehage et al., 2011). In young adults, such cognitive engagement during complex motor practice provides a plausible rationale for exploring associations between performance improvement and cognitive (Piper et al., 2015) or psychological variables (Harris et al., 2023) .

# 2. Objectives and hypotheses

## 2.1 Primary objective

To compare the effectiveness of two juggling training methods on juggling performance.

## 2.2 Secondary objectives

1. **Temporal evolution and order effect:** to describe and compare changes in juggling performance across the three assessment time points: baseline, Evaluation 1, and Evaluation 2. Performance will be compared between sequences (A→B vs B→A) to estimate differences attributable to exposure order.
2. **Associations with secondary and exploratory variables:** to explore whether changes in juggling performance are associated with: a) **Cognition:** (i) visuospatial ability/mental rotation, (ii) visuospatial working memory (spatial span), and (iii) response inhibition and sustained attention. b) **Handgrip Strength** c) **Questionnaires:** affect and flow.

## 2.3 Hypotheses

- **H1 (primary, superiority effect):** bouncing-based juggling (Method B) will result in greater performance gains compared to throwing-based juggling (Method A), particularly in the early stages of acquisition, due to the increased temporal window for feedback processing and motor planning. The method effect on performance will be estimated while accounting for period (1 vs 2) and sequence (A→B vs B→A) in the analytical model.
- **H2 (learning-order effect):** juggling performance and/or the magnitude of change between assessments will differ by exposure sequence (A→B vs B→A), reflecting an effect of learning order.
- **H3 (exploratory, cognition):** larger improvements in juggling performance will be associated with changes in cognitive performance.
- **H4 (exploratory, covariates/psychosocial):** handgrip strength, affective responses to juggling, and flow state during juggling practice will be positively associated with performance and/or its change, and may act as covariates or exploratory predictors.

# 3. Study design

## 3.1 Study type

This is a two-period, two-sequence intervention trial A→B and B→A with repeated measurements of the primary outcome at three time points: baseline, Evaluation 1, and Evaluation 2 (Wellek & Blettner, 2012).

The sequence will be assigned through cluster randomization at the class-group level, such that three class groups will follow the A→B sequence and three class groups will follow the B→A sequence (1:1 allocation). Within each class group, all students will receive the same sequence to minimize contamination (Campbell et al., 2012). The protocol follows established guidance for specifying essential design elements in trial protocols (A.-W. Chan et al., 2025).

The trial is designed within a superiority framework to test the hypothesis that Method B provides a more effective entry point for juggling acquisition than Method A. The primary analysis will estimate the method effect while accounting for sequence and period (Wellek & Blettner, 2012) and considering the hierarchical data structure (class-group clustering) (Johnson et al., 2015) and repeated measurements within students. As a complementary analysis, an estimate based solely on Period 1 will also be presented as a parallel-group comparison less influenced by prior exposure (Rietbergen & Moerbeek, 2011).

The trial was registered on ClinicalTrials.gov (Identifier: NCT07528924).

## 3.2 Rationale for the cross-over design

A two-period, two-sequence design is adopted for three main reasons:

1. **Efficient comparison of methods:** assessing the same participants across multiple time points improves precision in characterizing learning trajectories and reduces inter-individual variability in longitudinal follow-up (Hui et al., 2015).
2. **Order effect (central objective):** in motor learning tasks such as juggling, practice order may induce transfer or interference between methods. The AB-BA scheme enables estimation of differences attributable to starting with air-throwing (A) versus ground-bounce practice (B), both in performance trajectories and in the final level attained (Wellek & Blettner, 2012).
3. **Feasibility and educational equity:** in an academic setting, all students receive both training modalities during the study, supporting equitable learning opportunities (Rietbergen & Moerbeek, 2011).

Cluster allocation (class group) is justified for operational reasons and to reduce contamination (Campbell et al., 2012; Rietbergen & Moerbeek, 2011). Given this class-based design, potential intra-class correlation is anticipated (Hedges & Hedberg, 2013) and will be addressed analytically through an appropriate hierarchical framework (Johnson et al., 2015).

**Carryover considerations:** unlike pharmacological cross-over settings where washout may plausibly eliminate effects, behavioural interventions can show persistent carryover; therefore, Period 2 is interpreted primarily for order/transfer/interference, and Period 1 is emphasized for estimating the method effect without prior exposure (Kulnik et al., 2025; Shi & Ye, 2024). Accordingly:

- The protocol pre-specifies an analysis that includes period and sequence, interpreting Period 2 primarily as information on order-related effects and transfer between methods.
- As a robustness analysis and to obtain an estimand less influenced by prior exposure, the between-method comparison based on Period 1 will also be reported, which is equivalent to a parallel-group comparison between sequences prior to crossover (Rietbergen & Moerbeek, 2011).

## 3.3 Cluster structure

Six class groups (clusters) will be included. Three clusters will be assigned to the A→B sequence and three clusters to the B→A sequence (1:1 ratio). The class group will be the unit of allocation, and the student will be the unit of observation with repeated measures (Campbell et al., 2012).

## 3.4 Duration

The study comprises baseline assessment, Training Period 1, Evaluation 1, a 1-week wash-out interval, Training Period 2, and Evaluation 2. Each intervention period will last approximately 4 weeks, determined by the academic calendar and assessment windows. Total study duration is estimated at approximately 12 weeks.

## 3.5 Overall schedule

The full schedule is provided in Appendix VIII; a brief summary is as follows:

- **Week 0:** Baseline (Evaluation 0) and initial supervised session
- **Weeks 1–4:** Period 1 (intervention)
- **Week 5:** Evaluation 1
- **Week 6:** Separation interval (washout)
- **Weeks 7–10:** Period 2 (intervention)
- **Week 11:** Evaluation 2

# 4. Setting, participants, and recruitment

## 4.1 Setting

The study will be conducted within the “Juegos Motores” (*Motor Games)* course of the Bachelor’s degree in Physical Activity and Sport Sciences at the University of Granada during the 2025–2026 and 2026–2027 academic years. Scheduled practical sessions in the course will serve as the naturalistic setting for implementing the two juggling training modalities (A and B) across two sequential recruitment waves, while maintaining the usual teaching dynamics (Rogers et al., 2012) (Rogers et al., 2012). Both classes and assessments will take place at the “Toño Santos” Sports Hall of the Faculty of Sport Sciences.

## 4.2 Sample size

## The study aims to include a pragmatic sample of approximately 200 students recruited across two consecutive academic years. Detailed justification of the sample size, including power calculations and minimum detectable difference (MDD) based on Monte Carlo simulations (Martens et al., 2000), is provided in Section 15.

## 4.3 Inclusion criteria

Students will be eligible if they meet all of the following criteria:

- Enrolled in the *Motor Games* course.
- Aged ≥18 years and able to understand and provide informed consent.
- Provide explicit agreement to participate in the study assessments.

## 4.4 Exclusion criteria

Participants will be excluded if any of the following apply:

- Injury or medical condition that prevents safe participation in juggling, based on self-report prior to baseline assessment.
- Uncorrected or insufficiently corrected visual impairment that prevents seeing and tracking the balls during practice and assessments.
- Previous experience or high-level juggling proficiency (Geller et al., 2023)(e.g., students who self-identify as being in the improvement or expert stages, or those able to do a 3-ball cascade for >20 catches at baseline), to avoid ceiling effects.

## 4.5 Recruitment and Consent Procedure

The recruitment process will be integrated into the natural flow of the academic course to ensure high reach while maintaining the voluntary nature of the study. The ethical framework governing this process is detailed in Section 16.

Recruitment and Enrollment Potential participants will be recruited through an in-class presentation during the first week of the semester. During this session, the research team will explain the study’s objectives and the crossover design. Students will receive a digital Information Sheet via the institutional Virtual Campus. Informed consent will be obtained electronically via Google Forms prior to any research-specific data collection (e.g., initial questionnaires or cognitive batteries).

Distinction Between Academic and Research Activities A clear distinction will be maintained between mandatory academic requirements and voluntary research participation:

Academic requirement: All students enrolled in the course must record their juggling performance as part of their standard continuous evaluation and grading process (Yoo et al., 2009).

Research participation: Participation refers exclusively to authorizing the research team to use the derived data (performance scores, questionnaires, and video analysis) for scientific purposes.

The decision to participate or withdraw consent will have no impact on academic grading, teaching activities, or the provision of ECTS credits. No financial compensation will be provided.

Specific Consent for Video Data Given the sensitive nature of video recordings, the digital consent form will include a granular "opt-in" system. Participants can choose to authorize the use of their footage for: (i) internal research analysis only, (ii) educational purposes in classroom settings, or (iii) scientific dissemination in conferences or publications. Consent for any level may be revoked at any time, leading to the immediate deletion of the participant's data from the research database.

# 5. Randomization and allocation

## 5.1 Unit of randomization

The unit of randomization is the class group (cluster).

## 5.2 Generation of the random allocation sequence

Allocation of the six class groups to the two sequences (A→B and B→A) will be performed using a two-stage physical randomization procedure conducted by an individual external to both the research team and performance scoring (Pandis, 2011): Prof. Belén Feriche Fernández-Castanys (Dean of the Faculty of Sport Sciences, University of Granada).

First, a standard six-sided die will be rolled to determine which sequence will be assigned to the first three selected clusters. If the result is odd, the first three selected clusters will be assigned to sequence A→B; if the result is even, the first three selected clusters will be assigned to sequence B→A. The remaining three clusters will be automatically assigned to the alternate sequence, ensuring 1:1 allocation by clusters (3 vs 3) (Campbell et al., 2012).

Next, each class group will be pre-coded with a numerical identifier from 1 to 6 (C1–C6). The die will then be rolled repeatedly to select, without replacement, three distinct cluster codes. If a previously selected number occurs, the roll will be repeated until an unselected code is obtained. The three selected clusters will be assigned to the sequence determined in the first stage (A→B or B→A), and the remaining three clusters will be assigned to the other sequence.

The entire procedure will be video-recorded and documented in a randomization log (date, location, responsible person, decision rules, and final allocation).

**Restriction to avoid marked imbalance between sequences.** Because cluster sizes may differ, a re-randomization rule will be applied to avoid marked imbalance in the total number of students across sequences, using the number of eligible students per class group available at the time of randomization. Marked imbalance will be defined as a ratio >1.5 between the estimated total sample sizes of the two sequences (i.e., a difference >50%). If this condition is met, the allocation will be discarded and the entire randomization procedure repeated until an allocation meeting the criterion is obtained (Morgan & Rubin, 2012).

## 5.3 Allocation concealment, custody, and implementation

To minimize bias during initial assesment (McCoy, 2017), the sequence allocation will not be generated or disclosed until completion of the baseline assessment. The randomization video and log will be archived in the study custody folder. The final allocation table (class group → sequence) will be held by JF-O and will not be disclosed to other personnel involved in baseline assessments until baseline data collection is completed, thereby keeping the team blinded to each class group’s sequence during that period.

Allocation will be communicated to each class group after baseline, during the first subsequent class session, together with the schedule and materials for Period 1. Students will be asked not to share operational information about their assigned modality with students from other class groups to reduce between-cluster contamination (Keogh-Brown et al., 2007). Due to the nature of the intervention, students cannot be blinded to the training modality. However, performance scoring from video will remain blinded to the assigned sequence through file coding (anonymized ID and coded assessment timepoint only) and recording of scoring in an independent file by the assessor, which will later be merged into the research database by JF-O.

# 6. Interventions

## 6.1 Common components (both interventions)

The program comprises two 4-week intervention periods (Period 1 and Period 2) separated by a 1-week separation interval, in accordance with the cross-over design described above. In each period, participants are prescribed five sessions per week, with a target duration of 20 minutes per session (approximately 20 sessions per period, 40 sessions in total, and 800 minutes of planned practice). Additional juggling practice beyond that prescribed in the protocol will be explicitly discouraged; if undertaken, students will be asked to record the time spent (minutes) and the type of activity performed in order to document potential contamination or additional exposure.

Training will be primarily self-directed and supported by standardized official video tutorials (Yoo et al., 2009) that progress from lower to higher motor complexity in structured blocks, and by a per-session log (spreadsheet). In each session, students will practice the pattern/level corresponding to the active method’s tutorial (A or B, depending on the period), maintaining continuous practice for the target duration and taking brief breaks if needed. The goal is to progress to the next pattern once the pre-specified progression criterion is met. Within a given session, students may practice up to a maximum of three different patterns.

**Per-session log.** At minimum, students will record the date, session duration (minutes), active method (A or B according to the period), pattern(s)/level(s) practiced, and brief notes (optional; e.g., incidents or discomfort). A session will be considered valid when the student practices juggling for 20 minutes and records the session with the minimum required information. If no record exists for a planned session, that session will be treated as not completed for adherence calculations (Toval et al., 2024).

**Materials and setting (common).** Three frontennis balls (standard course equipment) will be used. Training may be conducted wherever the student prefers (e.g., home or university facilities), provided that a safe environment with sufficient space is ensured. For bouncing-based practice, a firm, stable, and uniform surface is recommended; for throwing-based practice, an obstacle-free area with sufficient vertical clearance is recommended.

Self-selected warm-up is proposed for the sessions given the technical-motor nature of the activity and the fixed 20-minute duration, following the guidance provided in the video tutorials.

**Progression criterion.** Students will advance to the next pattern when they achieve 20 consecutive successful catches in the current pattern (applicable to one-hand and two-hand patterns). A “successful catch” is defined as catching/receiving the ball with sufficient control to continue the pattern without a drop or interruption, consistent with the functional definition demonstrated in the course video tutorials (Iwatsuki & Regis, 2021).

Video tutorials will be provided through the official course repository (see S2 File).

## 6.2 Specific Training Modalities (Methods A and B)

Both interventions are identical in volume and progression structure, differing only in the task constraints. Intervention A (Throwing-based) utilizes traditional air-toss patterns, while Intervention B (Bouncing-based) utilizes floor-rebound patterns. Both methods follow the block-structured progression defined in the official tutorials.

## 6.3 Materials delivery and access

Video tutorials and materials will be distributed through the official course website. Given the cross-over design, each class group will follow the active method according to its assigned sequence (A→B or B→A) in each period. To reduce between-cluster contamination, reminders and organized access to the materials corresponding to the active method will be provided during each period, and students will be explicitly asked not to share materials across class groups while the study is ongoing.

## 6.4 Initial supervised session

A supervised introductory session will be held in Week 0 (60 minutes), delivered by JF-O and AF-R, to standardize: (i) how to use the video tutorials, (ii) how to structure the self-directed practice sessions, and (iii) how to record per-session adherence.

## 6.5 Discontinuation or modification of the intervention

Participants will be instructed to stop practicing and not continue the session if they experience pain, dizziness, significant discomfort, or any incident (e.g., upper-limb injury) (Common Terminology Criteria for Adverse Events, 2026). They will be asked to record the event in the session log and notify the responsible teaching staff. Incident management will follow the Faculty of Sport Sciences accident protocol (see Section 14).

# 7. Measures and Procedures

## 7.1 Assessment Time Points

Assessments will be conducted at three time points:

- **Baseline (pre–Period 1):** week of March 2, 2026 (Monday–Thursday sessions).
- **Evaluation 1 (post–Period 1):** April 8–14, 2026.
- **Evaluation 2 (post–Period 2):** May 20–26, 2026.

At all three time points, the same juggling assessment battery and identical operational procedures will be applied.

## 7.2 Juggling Assessment

### 7.2.1 Structure of the Assessment Battery

Juggling performance will be assessed using a comprehensive battery of tasks organized by support surface (feet on the floor vs. feet on a BOSU balance trainer) (Laudner & Koschnitzky, 2010; Moreno et al., 2023) and execution type (air throws vs. ground bounces) (Malik et al., 2022). To minimize systematic fatigue effects, the order of blocks (Floor vs. BOSU) will be counterbalanced across participants.

**Assesment Battery**

First, seven air-throw tasks will be evaluated:

1. One ball, right hand.
2. One ball, left hand.
3. Two balls in simultaneous columns (one ball per hand).
4. Two balls with alternating throws, switching hands.
5. Two-ball cycle with the right hand (inside-to-outside direction).
6. Two-ball cycle with the left hand (inside-to-outside direction).
7. Three-ball task using both hands.

Next, seven bounce tasks will be assessed under the same surface condition:

1. One ball, right-hand bounce.
2. One ball, left-hand bounce.
3. Two balls bouncing in simultaneous columns (one ball per hand).
4. Two balls with alternating bounce, switching hands.
5. Two-ball cycle with the right hand (outside-to-inside direction).
6. Two-ball cycle with the left hand (outside-to-inside direction).
7. Three-ball bounce task.

In addition to these “basic” blocks, six training-specific progression patterns will be assessed: *Rebound Active (RA), Rebound Passive (RB), Rebound Imperative (RC), Standard Cascade (A), Cascade with Cycle (C), and Successive Columns (CH).*

The same battery will be evaluated in stable (floor) and unstable (Bosu). In all tasks, the number of successful catches will be recorded (Moreau & Wiebels, 2021), applying the same attempt structure and stopping criteria described in Sections 7.2.3 and 7.2.4. For the cascade-with-cycle pattern, the default cycle direction will be inside-to-outside.

### 7.2.2 Operational Procedure

Multiple participants may be assessed simultaneously in separate spaces. An external assistant will follow a checklist and indicate task order to prevent omissions.

All performances will be video-recorded as part of standard grading procedures. For research purposes, only derived variables (catch counts) from participants who have provided informed consent will be extracted and analyzed.

For scoring quality control, intra-rater reliability will be evaluated in a random 10% subsample of videos, which will be rescored to detect systematic errors and document scoring consistency. Any relevant discrepancies will be resolved through video review and application of the standardized scoring manual.

### 7.2.3 Attempts and Warm-up

Participants will be allowed **two attempts per task**. Free warm-up will be permitted prior to the start of the evaluation.

### 7.2.4 Task Stopping Criteria

- **One-hand tasks:** maximum of 30 successful catches per attempt.
- **Two-hand tasks:** maximum of 60 successful catches per attempt.

Stopping criteria (30 one-hand catches; 60 two-hand catches) were chosen to prevent excessive fatigue while still capturing high-level proficiency. Based on expert-juggler work defining juggling proficiency as maintaining the pattern for ~20 s (Gerber et al., 2014), we used catch-count thresholds to facilitate scoring, with 30 successful catches serving as an approximate proxy for ~15 s. An attempt will terminate either upon reaching the maximum number of catches or upon a failed attempt (loss of ball control preventing a successful catch). No pauses are permitted within an attempt. After a failed attempt, the participant will immediately prepare for the second attempt. The total duration of the assessment session is estimated at 45 minutes, including mandatory 1-minute rest intervals between blocks to ensure recovery.

**Task scoring:** the best of the two attempts (highest number of valid catches) will be retained.

## 7.3 Demographic Covariates

The following variables will be collected:

- **Age (years):** calculated from recorded date of birth.
- **Sex at birth:** categorized as Male or Female.
- **Height:** in meters.
- **Weight:** in kilograms.
- **Laterality:** categorized as left or right.
- **Juggling previous experience**: Self-reported perceived skill level, 1-5 Likert scale, where 1 = total beginner and 5 = expert.

## 7.4 Questionnaires

Questionnaires will be administered at Baseline, Evaluation 1, and Evaluation 2 in Spanish via Google Forms. Participants will respond based on their experience during that specific session.

### 7.4.1 Positive and Negative Affect Schedule (PANAS)

The PANAS consists of 20 items with five response options (Never to Always) (Sandín et al., 1999).

### 7.4.2 Brief Inventory of Optimal Experiences (Flow)

Flow experience during the juggling activity will be assessed using the Brief Inventory of Optimal Experiences (Flow) (Calero & Injoque-Ricle, 2013), with a 5-point Likert scale.

## 7.5 Handgrip Strength

Handgrip strength will be assessed using digital dynamometry (TKK 5101 Grip D, Takey, Tokyo Japan).

The test will be performed in standing position, with the arm alongside the body and the dynamometer held unsupported, avoiding contact between the arm or device and the trunk or thigh during maximal effort.

Grip span will be standardized at 5.5 cm for males and 4.5 cm for females (sex at birth) and kept constant across measurements.

Each participant will perform two maximal attempts per hand, alternating hands. The value in kilograms (kg) will be recorded for each attempt. The primary variable will be the highest value obtained (best of two attempts) for each hand.

**Assessment time points:** Baseline and Evaluation 2.

## 7.6 Cognition

A brief computerized cognitive battery will be administered, comprising three tasks targeting domains plausibly involved in juggling learning:

1. **Attentional/Inhibitory control:** Go/No-Go Task.
2. **Visuospatial memory:** Matrix Rotation Task.
3. **Working memory:** Corsi Block-Tapping Test.

Tasks will be administered via the PEBL platform in a classroom setting (Piper et al., 2015), with each participant using their own laptop. The research team will coordinate synchronized start and end times. Participants will be instructed to complete tasks in full-screen mode and minimize interruptions. The use of personal headphones is recommended to provide acoustic isolation and minimize auditory distractions. However, listening to music or any other audio content is strictly prohibited to ensure that cognitive resources are fully directed toward the tasks. Any violation of this rule will result in the invalidation of that specific assessment session.

**Standardized order and approximate duration (25 minutes total):**

1. Go/No-Go (7 minutes).
2. Matrix Rotation (10 minutes).
3. Corsi forward (8 minutes).

**Recorded outcomes:**

- **Go/No-Go:** commission errors (primary), omission errors, and reaction time in correct Go trials (recommended metric: median).
- **Matrix Rotation:** accuracy (% correct) and reaction time in correct trials (median).
- **Corsi forward:** maximum span and total score (standard task output).

**Assessment time points:** Baseline and Evaluation 2.

# 8. Definition of Outcome Variables

## 8.1 Task-Level Variables and normalized scores

For each task included in the juggling battery, the number of successful catches from the best of two attempts will be recorded. Higher catches indicate superior performance.

- **One-hand tasks:** range 0–30 successful catches per attempt.
- **Two-hand tasks:** range 0–60 successful catches per attempt.

To enable comparability across tasks with different maximum values, each task will be converted to a common 0–30-point scale:

- **One-hand tasks (maximum 30 catches):** Normalized score = number of successful catches in the best attempt (0–30).
- **Two-hand tasks (maximum 60 catches):** Normalized score = catches / 2 (0–30).

## 8.2 Primary Outcome: Global Composite Score

Three composite scores will be defined based on the normalized task-level scores (0–30 points; see Section 8.2).

Within each support surface condition, the same tasks will be included: 14 “basic” tasks (7 air-throw and 7 ground-bounce tasks), plus 6 training progression patterns (RA, RB, RC, A standard cascade, C cascade with cycle, and CH successive columns), resulting in a total of 20 tasks per surface. Accordingly:

- Stable score (feet on the floor): sum of the 20 normalized task scores performed on the floor (theoretical range: 0–600 points).
- Unstable score (feet on BOSU): sum of the same 20 normalized task scores performed on the BOSU (theoretical range: 0–600 points).

The primary study outcome will be the Global score, defined as the sum of Stable score and Unstable_score (40 tasks in total; theoretical range: 0–1200 points). Task composition will be identical at Baseline, Evaluation 1, and Evaluation 2.

If a participant fails to complete a specific task due to absence or technical error, a second assessment opportunity will be scheduled within the same evaluation window. If the task remains uncompleted after this second attempt, a score of 0 will be assigned for that task to maintain a conservative estimate of performance gains.

Missing Data Policy: If a task remains uncompleted after a second opportunity, a score of 0 will be assigned.

## 8.3 Secondary and Exploratory Non-Motor Variables

### 8.3.1 PANAS

Positive Affect (PA) and Negative Affect (NA) will be calculated as the mean of their items (range 1–5). For PA, higher scores indicate greater positive affect. For NA, higher scores reflect greater negative affect (disaffection/rejection), while lower scores are considered more favorable (Sandín et al., 1999). The derived variables will be two, one for PA and other one for NA, they Will be calculated of the mean of their ten items.

*Missing items:* Scores will be computed if no more than one item is missing per subscale, using mean imputation.

### 8.3.2 Flow (Brief Inventory of Optimal Experiences)

Flow will be quantified as the mean of its nine items (range 1–5). Higher scores indicate a greater flow state (Calero & Injoque-Ricle, 2013).

The primary derived variable will be the total score, calculated as the mean of the nine items (range 1–5). Scores will be computed if no more than one item is missing, using mean imputation.

### 8.3.3 Cognition (Baseline and Evaluation 2)

Outcomes include:

- **Attentional control (Go/No-Go):** Commission errors (primary), omission errors, and median reaction time (RT). Lower values in errors and RT indicate better performance.
- **Visuospatial memory** **(Matrix Rotation):** Accuracy (% correct; higher is better) and median RT (lower is better).
- **Working memory (Corsi forward):** Maximum span and total score (higher is better).

### 8.3.4 Handgrip Strength (Baseline and Evaluation 2)

- **Handgrip strength (kg):** defined as the highest value obtained from two maximal attempts per hand. Higher values indicate greater isometric strength.

# 9. Covariates and Complementary Measures for the Analysis

To adjust for individual differences and potential confounding factors, the following variables will be included in the analytical models:

**Demographic covariates:** age and sex at birth (as defined in the data collection section).

**Baseline physical and cognitive status:** Handgrip strength and cognitive battery performance (Go/No-Go, Matrix Rotation, and Corsi span).

**Questionnaires:** PANAS (Positive and Negative Affect) and Flow scores (Brief Inventory of Optimal Experiences) collected at all three time points.

The available sample size for each measure and analysis will be explicitly reported. The inclusion of these variables as covariates aims to increase the precision of the method effect estimate and to explore potential interactions between learner profiles and training outcomes.

# 10. Attendance, Compliance, and Adherence

## 10.1 Recording Procedures

Attendance and compliance will be monitored using an online spreadsheet (Appendix III), in which the following will be recorded for each session: date, minutes of practice, and tasks/patterns performed (with optional brief comments).

The unit of recording will be the planned session (5 sessions per week; 20-25 sessions per period). Metrics will be reported per period and globally, and will be descriptively compared between sequences.

## 10.2 Operational Definitions

**Attendance:** A session is "attended" if date and minutes are recorded, regardless of duration.

**Compliance:** An attended session is "compliant" when the recorded duration is ≥20 minutes.

**Adherence:** The primary metric of program exposure, calculated as the percentage of planned sessions (out of total per period) that are both attended and compliant.

## 10.3 Per-Protocol (PP) Definition

The primary analysis will follow an intention-to-treat (ITT) principle, including all participants regardless of their level of engagement (McCoy, 2017). To complement this, a per-protocol (PP) analysis will be conducted to estimate the effect under optimal conditions (De Souza et al., 2016).

The PP population is defined as participants who achieve an adherence rate of at least 60% (≥12 compliant sessions) in each period. This threshold is established as a dosage likely to induce stable changes in bimanual coordination for complex motor skills (Sampaio-Baptista et al., 2015)**.**

# 11. Blinding and Contamination Control

## 11.1 Blinding

Juggling performance scoring will be conducted by AF-R based on video recordings, using only an anonymized study identifier. AF-R will not have access to the main research dataset.

Videos will be exported and stored in a restricted-access working folder. A “blinded” copy will be generated for scoring purposes and renamed using a standardized scheme that includes only the study ID and assessment time point, without reference to class group, sequence, or period. Preparation and renaming of files for scoring will be performed by JF-O prior to sharing them with AF-R. In the event of accidental unblinding, the file will be re-coded and the incident will be documented.

To minimize bias, videos will follow a neutral labeling scheme (anonymized ID and coded assessment time point only), avoiding any indication of group or sequence in file names. AF-R will record scores in an independent Excel file. Subsequently, JF-O will integrate these scores into the main dataset using the anonymized study ID.

Given that the nature of the intervention does not allow blinding of students, teaching staff of the course (except JF-O) will remain blinded to the sequence assigned to each class group whenever feasible.

## 11.2 Contamination

Contamination between class groups will be minimized through:

- Separate distribution of materials by class group and period.
- Explicit instructions not to share training content or guidelines across groups.
- Reminders during intervention periods.

Any juggling practice beyond the prescribed intervention will be discouraged. Participants will be explicitly requested to refrain from teaching techniques to peers in different class groups until the completion of the trial. Such additional practice will be recorded (minutes and type of practice) to document extra exposure and, if appropriate, to consider it in sensitivity analyses.

# 12. Data Management, Roles, and Security

## 12.1 Identification and Anonymization

To ensure maximum participant privacy while maintaining academic functionality, a dual-layer anonymization system will be used:

**Anonymized Study ID:** A structured alphanumeric code (e.g., GR_6123) based on group enrollment order.

**Unique Avatar:** A secondary pseudonym consisting of a standardized combination of a playing card and a predefined object (e.g., "Q-Diamond-Fire"). This system generates 240 unique combinations, allowing students to identify their records in public logs without revealing personal identity

## 12.2 Storage and Access

Data will be stored on an institutional cloud platform compliant with the General Data Protection Regulation (GDPR) and national legislation (LOPDGDD).

i) Folder A (Master Key): Restricted to JF-O.
ii) Folder B (Anonymized Dataset): Restricted to JF-O.
iii) Folder C (Videos/Scoring): Access granted to AF-R and JF-O.

## 12.3 Roles

- Generation and custody of study IDs and master key: JF-O.
- Management of research dataset and file integration: JF-O.
- Video recording and logistical coordination of assessments: AF-R.
- Scoring of juggling performance (video-based): AF-R.

## 12.4 Video Management and Dissemination

Juggling assessment performances will be recorded by AF-R with specific informed consent for recording and dissemination. Video recording forms part of the standard academic evaluation procedure.

For research purposes, only derived data from videos of participants who have provided research consent will be extracted and analyzed.

In all cases, videos will not include names, surnames, study IDs, avatars, or any other personal identifiers in the video content, file name, description, or associated metadata.

# 13. Statistical Analysis Plan

## 13.1 General Principles

All analyses will be conducted using R (version 4.5.3 or higher). The core modeling will be performed using the LMMstar package for linear mixed models, with emmeans for the extraction of marginal means and post-hoc comparisons. Mediation analyses will be executed via the bruceR::PROCESS wrapper. Data manipulation and visualization will rely on the tidyverse, ggtext, and sjPlot ecosystems. Statistical significance is set at α = 0.05.

The primary analysis will follow an intention-to-treat (ITT) approach, maintaining participants in the sequence assigned to their class group and using all available data. Because randomization is performed at the class-group level, all models will account for the hierarchical structure (students nested within 6 class groups). Given the small number of clusters, inference methods with small-sample corrections will be applied, and findings will be accompanied by sensitivity analyses.

## 13.2 Population and Data Pre-processing

**Analysis Populations:** The primary analysis will follow an Intention-to-Treat (ITT) approach. All participants will be analyzed according to their original cluster-randomized sequence, regardless of adherence, using all available data points.

**Standardization:** To allow comparison across heterogeneous motor and cognitive scales, variables will be Z-standardized using the mean and standard deviation of the Baseline (Pre) assessment as the reference distribution. This ensures that observed changes are interpreted in units of baseline variation.

**Subgroup Categorization:** For exploratory moderator analysis, participants will be stratified into "high" vs. "low" initial performers using a median-split based on baseline performance.

## 13.3 Primary Outcome Analysis

Due to the inherent nature of motor learning, carryover effects are expected. Therefore, the unbiased efficacy of the training methods (Air vs. Floor) will be estimated using Period 1 data. Given the hierarchical structure with a small number of clusters (k=6), we will implement a two-stage covariate-adjusted cluster-level analysis to prevent Type I error inflation (Wellek & Blettner, 2012), where prior exposure to the alternative method cannot occur.

- Stage 1 (Individual level): A linear regression will model the Evaluation 1 Global Composite Score (GCS) adjusted for the Baseline GCS.
- Stage 2 (Cluster level): The resulting residuals will be averaged by cluster. These cluster-mean residuals will be compared between methods (A vs. B) using an independent t-test with 4 degrees of freedom (k - 2).
- Effect Size: Results will be reported as mean differences in GCS points with 95% Confidence Intervals (CI).

## 13.4 Secondary Analysis: Crossover Dynamics and Transfer

To explore the full crossover design, a longitudinal LMM will be fitted including both periods. Model Structure: Fixed effects will include *Method* (A vs. B), *Period* (1 vs. 2), and *Sequence* (A→B vs. B→A). A random intercept for participants will account for within-subject correlation. Period 2 Decomposition: Period 2 performance will be analyzed through two lenses: i) *Total Learning*: Adjusted for Baseline, representing the cumulative effect of both methods. ii) *Period Gain*: Adjusted for Evaluation 1 (Pre-Period 2), quantifying the specific gain in the second block and identifying potential asymmetric transfer or contextual interference.

## 13.5 Mediation and Mechanistic Models

To investigate *why* a method might be superior, mediation models (5,000 bootstrap iterations) will be constructed:

**Path A:** Effect of Training Method on the change in mediators (e.g., Flow State, Attentional Control).

**Path B:** Association between mediator change and post-intervention juggling performance, adjusting for baseline.

**Indirect Effect:** Estimation of the proportion of the training effect explained by psycho-cognitive shifts.

## 13.6 Baseline Profiling and Predictive Models

Cross-sectional associations at baseline will characterize the "juggling-ready" profile:

**Standardized Predictors:** Multiple regressions will use standardized Betas to compare the relative weight of physical (Handgrip) vs. cognitive variables.

**Model Comparison:** We will contrast a "Physical Dimension" model against a "Psycho-cognitive Dimension" model using R^2^ to determine which domain better explains baseline motor skill.

## 13.7 Missing Data and Sensitivity Analyses

**Missing Data:** Longitudinal models will utilize Full Information Maximum Likelihood (FIML), allowing participants with missing observations to contribute to the estimation without bias, assuming data are Missing at Random (MAR).

**Sensitivity Analyses:** To ensure the stability of the findings, we will perform:

**Per-Protocol Analysis:** Excluding participants with <60% attendance.

**Outlier Impact:** Excluding "expert" outliers (Baseline GCS > 20).

**Inference Validation:** Comparing the two-stage cluster analysis against Cluster-Robust Standard Errors (CRSE) with small-sample corrections.

# 14. Adverse Events

In accordance with safety standards for exercise interventions, any incident, accident, or discomfort occurring during study activities will be managed under the official Accident Response Protocol of the Faculty of Sport Sciences. An adverse event (AE) is defined as any undesirable occurrence, including pain, injury, dizziness, falls, or significant discomfort, requiring the cessation of activity (based on Common Terminology Criteria for Adverse Events). Given the postural demands of the intervention, specific monitoring will be applied to tasks involving unstable surfaces (BOSU). AEs will be recorded by the research team (evaluations) or through participant self-reports (training sessions). For each event, the log will document: (i) date/time, (ii) description and severity, (iii) assistance required, (iv) relationship to the study, and (v) resolution status. Safety stopping criteria: practice will be immediately discontinued if a participant reports acute pain, dizziness, blurred vision, or marked instability. All AEs will be reported in aggregate form in the final manuscript.

# 15. Sample Size and Justification

The planned sample consists of the full cohort of students enrolled in the 2025–2026 and 2026–2027 academic years (~200 students in 6 class groups). While this is a pragmatic study, the sample size was validated using Monte Carlo simulation based on a Minimum Detectable Difference (MDD) framework. Under conservative assumptions (6 clusters, ICC = 0.03, baseline-post correlation r = 0.50, and an assumed SD of 20 points for the 6-task composite), a sample of ~200 participants provides ~80% power to detect method differences of 10–11 points. To detect a minimally relevant difference of 12 points (representing ~2 points per task), the required sample is 155 participants with complete data. Accounting for a predicted 5% dropout rate, a minimum recruitment of 163 students is targeted. This calculation ensures that the study is sufficiently powered to address the primary objective during Period 1, even before accounting for the additional statistical power gained from the repeated-measures structure of the full crossover design.

# 16. Ethical Considerations

This study will be conducted in accordance with the Declaration of Helsinki and was approved by the Ethics Committee on Research of the University of Granada (Ref: 5654/CEIH/2026). Participation is entirely voluntary, and all students must provide electronic informed consent via Google Forms prior to any research-related data collection. Participants are informed of their right to withdraw from the study at any time without any academic or personal penalty. Data will be processed in a pseudonymized form to ensure confidentiality, in compliance with the General Data Protection Regulation (GDPR) and national legislation (LOPDGDD). The research team ensures a clear distinction between mandatory academic activities (video recording for grading) and voluntary research participation (use of data for scientific purposes), ensuring that the decision to participate has no impact on academic standing or grading.

# 17. Dissemination and Publication Plan

Results will be disseminated through conference presentations and publications in peer-reviewed scientific journals. In all cases, results will be presented in aggregate, non-identifiable form.

After study completion and/or primary publication, participants will be offered a summary of results in non-technical language through the usual course communication channels, without inclusion of individual-level data.

## 17.1 Authorship Criteria and Contributions (ICMJE)

Authorship of publications arising from this project will follow the criteria of the International Committee of Medical Journal Editors (ICMJE).

Individuals will qualify as authors only if they meet all criteria:

- Substantial contribution to study design or to data acquisition, analysis, or interpretation;
- Drafting the manuscript or critically revising it with important intellectual contributions;
- Approval of the final version;
- Agreement to be accountable for the integrity and accuracy of the work.

Contributions will be documented prospectively during the project, including, when useful, description using the CRediT taxonomy, to facilitate transparent decisions regarding authorship and order.

Individuals providing partial contributions (e.g., logistical or teaching support) will be acknowledged unless they meet ICMJE criteria. Disagreements regarding authorship or order will be resolved through documented discussion within the research team and, if unresolved, escalated to project coordination for a final decision based on the stated criteria.

# 18. Data Sharing Plan

The R analysis code and variable dictionary will be shared upon project completion via an institutional repository (e.g., Digibug) or equivalent version-controlled platform.

Following primary publication, pseudonymized individual-level data will be available upon reasonable request for research purposes. Shared materials will include the final dataset required to reproduce the analyses, the variable dictionary, and the analysis code.

The identity–ID correspondence table and any material allowing re-identification will not be shared. Data access will require a signed data use agreement prohibiting re-identification, limiting use to scientific purposes, and requiring appropriate citation of the study and repository.

In all cases, only aggregated and non-identifiable results will be publicly disseminated.

# 19. Discussion

This protocol describes a pragmatic, education-based trial that compares two juggling practice methods (air throws vs. ground bounces) and, secondarily, examines whether the order of exposure (A→B vs. B→A) shapes the trajectory of performance across repeated evaluations. The rationale is grounded in motor-learning evidence showing that practice schedule and practice order (e.g., blocked vs. random, and how interference is manipulated across sessions) can influence skill retention and transfer, with contextual interference as a central explanatory mechanism (Buszard et al., 2017; Czyż, Wójcik, & Solarská, 2024; Porter & Magill, 2010).

Juggling is a suitable model because complex coordinative skills often show heterogeneous and non-linear learning patterns. The use of a high-frequency longitudinal design (Baseline, Evaluation 1, Evaluation 2) allows us to capture these trajectories, moving beyond traditional pre-post snapshots that often miss the critical convergence or divergence points of different instructional methods (Geller et al., 2023). Furthermore, our composite score, spanning stable and unstable conditions, operationalizes a practical form of transfer by probing performance under altered postural constraints (Laudner & Koschnitzky, 2010).

A central methodological issue is carryover. Unlike pharmacological trials, motor-learning gains persist; therefore, the one-week interval is a scheduling separator rather than a mechanism to eliminate prior learning. While a one-week washout is insufficient to forget the skill, it serves to dissipate transient fatigue and resets the cognitive set for the new block of instructions. Accordingly, the primary estimand of the between-method effect is anchored to Period 1, while Period 2 is interpreted to characterize order-related phenomena, facilitation, or interference between methods (Wellek & Blettner, 2012).

Beyond motor performance, the inclusion of affect and flow scales (PANAS and Flow State Scale) helps contextualize intraindividual fluctuations and interindividual differences. Conceptually, flow reflects an adaptive coupling between challenge and skill; deviations in flow may help interpret departures from expected learning curves under different instructional constraints (Harris et al., 2023). Cognitive and strength measures remain exploratory, aimed at characterizing predictors of overall improvement rather than attributing changes specifically to one method (Voelcker-Rehage et al., 2011).

Key strengths include high ecological validity, standardized instructional content, and blinded video-based scoring. Acknowledging the pragmatic nature of the trial, participants cannot be blinded to the training modality. However, the risk of cross-group contamination is mitigated by the cluster-based allocation and the use of separate practice logs. These constraints justify our conservative interpretation focused on effect estimation and sensitivity analyses (De Souza et al., 2016; Huang et al., 2016).

## Educational Implications

If meaningful differences between methods or exposure order are observed, findings could inform how juggling progressions are sequenced in physical education professional training, including whether early emphasis on a given constraint set yields more robust performance across contexts. Reporting should emphasize transparent intervention description to support replication and curriculum translation.

## Future Research

Future work should include a delayed retention test beyond course completion to better separate transient performance gains from learning stability and to strengthen claims about transfer. Replication in other educational levels and age ranges is also warranted given evidence that learning responses to practice organization can depend on learner characteristics. Methodologically, increasing the number of clusters or adopting designs and inferential approaches optimized for educational cluster trials would improve robustness. Finally, studies could incorporate more comprehensive cognitive batteries and, where feasible, neurobiological measures with adequate sample sizes and rigorous controls.

# 20. References

Brydges, R., Carnahan, H., Backstein, D., & Dubrowski, A. (2007). Application of Motor Learning Principles to Complex Surgical Tasks: Searching for the Optimal Practice Schedule. *Journal of Motor Behavior*, *39*(1), 40-48. https://doi.org/10.3200/JMBR.39.1.40-48

Buszard, T., Reid, M., Krause, L., Kovalchik, S., & Farrow, D. (2017). Quantifying Contextual Interference and Its Effect on Skill Transfer in Skilled Youth Tennis Players. *Frontiers in Psychology*, *8*, 1931. https://doi.org/10.3389/fpsyg.2017.01931

Calero, A., & Injoque-Ricle, I. (2013). Propiedades psicométricas del Inventario Breve de Experiencias Óptimas (Flow). *Revista Evaluar*, *13*(1). https://doi.org/10.35670/1667-4545.v13.n1.6796

Campbell, M. K., Piaggio, G., Elbourne, D. R., Altman, D. G., & for the CONSORT Group. (2012). Consort 2010 statement: Extension to cluster randomised trials. *BMJ*, *345*(sep04 1), e5661-e5661. https://doi.org/10.1136/bmj.e5661

Chan, A.-W., Boutron, I., Hopewell, S., Moher, D., Schulz, K. F., Collins, G. S., Tunn, R., Aggarwal, R., Berkwits, M., Berlin, J. A., Bhandari, N., Butcher, N. J., Campbell, M. K., Chidebe, R. C. W., Elbourne, D. R., Farmer, A. J., Fergusson, D. A., Golub, R. M., Goodman, S. N., … Hróbjartsson, A. (2025). SPIRIT 2025 statement: Updated guideline for protocols of randomized trials. *Nature Medicine*, *31*(6), 1784-1792. https://doi.org/10.1038/s41591-025-03668-w

Chan, J. S. Y., Luo, Y., Yan, J. H., Cai, L., & Peng, K. (2015). Children’s age modulates the effect of part and whole practice in motor learning. *Human Movement Science*, *42*, 261-272. https://doi.org/10.1016/j.humov.2015.06.002

Czyż, S. H., Wójcik, A. M., & Solarská, P. (2024). The effect of contextual interference on transfer in motor learning—A systematic review and meta-analysis. *Frontiers in Psychology*, *15*, 1377122. https://doi.org/10.3389/fpsyg.2024.1377122

Czyż, S. H., Wójcik, A. M., Solarská, P., & Kiper, P. (2024). High contextual interference improves retention in motor learning: Systematic review and meta-analysis. *Scientific Reports*, *14*(1), 15974. https://doi.org/10.1038/s41598-024-65753-3

De Souza, R. J., Eisen, R. B., Perera, S., Bantoto, B., Bawor, M., Dennis, B. B., Samaan, Z., & Thabane, L. (2016). Best (but oft-forgotten) practices: Sensitivity analyses in randomized controlled trials. *The American Journal of Clinical Nutrition*, *103*(1), 5-17. https://doi.org/10.3945/ajcn.115.121848

Dudley, D., Mackenzie, E., Van Bergen, P., Cairney, J., & Barnett, L. (2022). What Drives Quality Physical Education? A Systematic Review and Meta-Analysis of Learning and Development Effects From Physical Education-Based Interventions. *Frontiers in Psychology*, *13*, 799330. https://doi.org/10.3389/fpsyg.2022.799330

Geller, N., Moringen, A., & Friedman, J. (2023). Learning juggling by gradually increasing difficulty vs. Learning the complete skill results in different learning patterns. *Frontiers in Psychology*, *14*, 1284053. https://doi.org/10.3389/fpsyg.2023.1284053

Gerber, P., Schlaffke, L., Heba, S., Greenlee, M. W., Schultz, T., & Schmidt-Wilcke, T. (2014). Juggling revisited—A voxel-based morphometry study with expert jugglers. *NeuroImage*, *95*, 320-325. https://doi.org/10.1016/j.neuroimage.2014.04.023

Harris, D. J., Allen, K. L., Vine, S. J., & Wilson, M. R. (2023). A systematic review and meta-analysis of the relationship between flow states and performance. *International Review of Sport and Exercise Psychology*, *16*(1), 693-721. https://doi.org/10.1080/1750984X.2021.1929402

Hedges, L. V., & Hedberg, E. C. (2013). Intraclass Correlations and Covariate Outcome Correlations for Planning Two- and Three-Level Cluster-Randomized Experiments in Education. *Evaluation Review*, *37*(6), 445-489. https://doi.org/10.1177/0193841X14529126

Hoffmann, T. C., Glasziou, P. P., Boutron, I., Milne, R., Perera, R., Moher, D., Altman, D. G., Barbour, V., Macdonald, H., Johnston, M., Lamb, S. E., Dixon-Woods, M., McCulloch, P., Wyatt, J. C., Chan, A.-W., & Michie, S. (2014). Better reporting of interventions: Template for intervention description and replication (TIDieR) checklist and guide. *BMJ*, *348*(mar07 3), g1687-g1687. https://doi.org/10.1136/bmj.g1687

Huang, S., Fiero, M. H., & Bell, M. L. (2016). Generalized estimating equations in cluster randomized trials with a small number of clusters: Review of practice and simulation study. *Clinical Trials*, *13*(4), 445-449. https://doi.org/10.1177/1740774516643498

Hui, D., Zhukovsky, D. S., & Bruera, E. (2015). Which Treatment Is Better? Ascertaining Patient Preferences With Crossover Randomized Controlled Trials. *Journal of Pain and Symptom Management*, *49*(3), 625-631. https://doi.org/10.1016/j.jpainsymman.2014.11.294

Iwatsuki, T., & Regis, C. J. (2021). Relatively Easy Criteria for Success Enhances Motor Learning by Altering Perceived Competence. *Perceptual and Motor Skills*, *128*(2), 900-911. https://doi.org/10.1177/0031512520981237

Johnson, J. L., Kreidler, S. M., Catellier, D. J., Murray, D. M., Muller, K. E., & Glueck, D. H. (2015). Recommendations for choosing an analysis method that controls Type I error for unbalanced cluster sample designs with Gaussian outcomes. *Statistics in Medicine*, *34*(27), 3531-3545. https://doi.org/10.1002/sim.6565

Keogh-Brown, M., Bachmann, M., Shepstone, L., Hewitt, C., Howe, A., Ramsay, C., Song, F., Miles, J., Torgerson, D., Miles, S., Elbourne, D., Harvey, I., & Campbell, M. (2007). Contamination in trials of educational interventions. *Health Technology Assessment*, *11*(43). https://doi.org/10.3310/hta11430

Kulnik, S. T., Carrozzo, A. E., & Crutzen, R. (2025). Persistent carry-over in a two-period randomised crossover design for behavioural interventions without the expectation of return to baseline after intervention cessation. *Trials*, *26*(1), 561. https://doi.org/10.1186/s13063-025-09358-9

Latino, F., Cataldi, S., & Fischetti, F. (2021). Effects of a Coordinative Ability Training Program on Adolescents’ Cognitive Functioning. *Frontiers in Psychology*, *12*, 620440. https://doi.org/10.3389/fpsyg.2021.620440

Laudner, K. G., & Koschnitzky, M. M. (2010). Ankle muscle activation when using the Both Sides Utilized (BOSU) balance trainer. *Journal of Strength and Conditioning Research*, *24*(1), 218-222. https://doi.org/10.1519/JSC.0b013e3181c490d4

Lorås, H. (2020). The Effects of Physical Education on Motor Competence in Children and Adolescents: A Systematic Review and Meta-Analysis. *Sports*, *8*(6), 88. https://doi.org/10.3390/sports8060088

Magelssen, C., Haugen, P., Reid, R., & Gilgien, M. (2022). Is there a contextual interference effect for sub-elite alpine ski racers learning complex skills? *Frontiers in Bioengineering and Biotechnology*, *10*, 966041. https://doi.org/10.3389/fbioe.2022.966041

Malik, J., Stemplewski, R., & Maciaszek, J. (2022). The Effect of Juggling as Dual-Task Activity on Human Neuroplasticity: A Systematic Review. *International Journal of Environmental Research and Public Health*, *19*(12), 7102. https://doi.org/10.3390/ijerph19127102

Martens, H., Dijksterhuis, G. B., & Byrne, D. V. (2000). Power of experimental designs, estimated by Monte Carlo simulation. *Journal of Chemometrics*, *14*(5-6), 441-462. https://doi.org/10.1002/1099-128X(200009/12)14:5/6%3C441::AID-CEM644%3E3.0.CO;2-F

McCoy, E. (2017). Understanding the Intention-to-treat Principle in Randomized Controlled Trials. *Western Journal of Emergency Medicine*, *18*(6), 1075-1078. https://doi.org/10.5811/westjem.2017.8.35985

Moreau, D., & Wiebels, K. (2021). Assessing Change in Intervention Research: The Benefits of Composite Outcomes. *Advances in Methods and Practices in Psychological Science*, *4*(1), 2515245920931930. https://doi.org/10.1177/2515245920931930

Moreno, F. J., Barbado, D., Caballero, C., Urbán, T., & Sabido, R. (2023). Variations induced by the use of unstable surface do not facilitate motor adaptation to a throwing skill. *PeerJ*, *11*, e14434. https://doi.org/10.7717/peerj.14434

Morgan, K. L., & Rubin, D. B. (2012). Rerandomization to improve covariate balance in experiments. *The Annals of Statistics*, *40*(2). https://doi.org/10.1214/12-AOS1008

Pandis, N. (2011). Randomization. Part 1: Sequence generation. *American Journal of Orthodontics and Dentofacial Orthopedics*, *140*(5), 747-748. https://doi.org/10.1016/j.ajodo.2011.06.020

Pauwels, L., Swinnen, S. P., & Beets, I. A. M. (2014). Contextual Interference in Complex Bimanual Skill Learning Leads to Better Skill Persistence. *PLoS ONE*, *9*(6), e100906. https://doi.org/10.1371/journal.pone.0100906

Piper, B. J., Mueller, S. T., Geerken, A. R., Dixon, K. L., Kroliczak, G., Olsen, R. H. J., & Miller, J. K. (2015). Reliability and validity of neurobehavioral function on the Psychology Experimental Building Language test battery in young adults. *PeerJ*, *3*, e1460. https://doi.org/10.7717/peerj.1460

Porter, J. M., & Magill, R. A. (2010). Systematically increasing contextual interference is beneficial for learning sport skills. *Journal of Sports Sciences*, *28*(12), 1277-1285. https://doi.org/10.1080/02640414.2010.502946

Renshaw, I., & Chow, J.-Y. (2019). A constraint-led approach to sport and physical education pedagogy. *Physical Education and Sport Pedagogy*, *24*(2), 103-116. https://doi.org/10.1080/17408989.2018.1552676

Rietbergen, C., & Moerbeek, M. (2011). The Design of Cluster Randomized Crossover Trials. *Journal of Educational and Behavioral Statistics*, *36*(4), 472-490. https://doi.org/10.3102/1076998610379136

Rogers, D. L., Kranz, P. L., & Ferguson, C. J. (2012). A Strategy for Involving Undergraduates in Research. *Journal of Hispanic Higher Education*, *11*(1), 55-66. https://doi.org/10.1177/1538192711435558

Sampaio-Baptista, C., Filippini, N., Stagg, C. J., Near, J., Scholz, J., & Johansen-Berg, H. (2015). Changes in functional connectivity and GABA levels with long-term motor learning. *NeuroImage*, *106*, 15-20. https://doi.org/10.1016/j.neuroimage.2014.11.032

Sandín, B., Chorot, P., Lostao, L., Joiner, T. E., Santed-Germán, M. Á., & Valiente-García, R. M. (1999). Escalas panas de afecto positivo y negativo: Validación factorial y convergencia transcultural. *Psicothema*, *11*(1), 37-51.

Shi, D., & Ye, T. (2024). *Behavioral Carry-Over Effect and Power Consideration in Crossover Trials* (arXiv:2302.01246). arXiv. https://doi.org/10.48550/arXiv.2302.01246

Telford, R. M., Olive, L. S., Keegan, R. J., Keegan, S., & Telford, R. D. (2021). Teacher and school outcomes of the Physical Education and Physical Literacy (PEPL) approach: A pragmatic cluster randomised controlled trial of a multicomponent intervention to improve physical literacy in primary schools. *Physical Education and Sport Pedagogy*, *26*(1), 79-96. https://doi.org/10.1080/17408989.2020.1799965

Toval, A., Solis-Urra, P., Bakker, E. A., Sánchez-Aranda, L., Fernández-Ortega, J., Prieto, C., Alonso-Cuenca, R. M., González-García, A., Martín-Fuentes, I., Fernandez-Gamez, B., Olvera-Rojas, M., Coca-Pulido, A., Bellón, D., Sclafani, A., Sanchez-Martinez, J., Rivera-López, R., Herrera-Gómez, N., Peñafiel-Burkhardt, R., López-Espinosa, V., … Ortega, F. B. (2024). Exercise and brain health in patients with coronary artery disease: Study protocol for the HEART-BRAIN randomized controlled trial. *Frontiers in Aging Neuroscience*, *16*, 1437567. https://doi.org/10.3389/fnagi.2024.1437567

Voelcker-Rehage, C., Godde, B., & Staudinger, U. M. (2011). Cardiovascular and Coordination Training Differentially Improve Cognitive Performance and Neural Processing in Older Adults. *Fronties in Human Neuroscience*, *5*. https://doi.org/10.3389/fnhum.2011.00026

Wellek, S., & Blettner, M. (2012). On the Proper Use of the Crossover Design in Clinical Trials. *Deutsches Ärzteblatt international*. https://doi.org/10.3238/arztebl.2012.0276

Wu, W. F. W., Young, D. E., Schandler, S. L., Meir, G., Judy, R. L. M., Perez, J., & Cohen, M. J. (2011). Contextual interference and augmented feedback: Is there an additive effect for motor learning? *Human Movement Science*, *30*(6), 1092-1101. https://doi.org/10.1016/j.humov.2011.02.004

Yoo, M. S., Son, Y. J., Kim, Y. S., & Park, J. H. (2009). Video-based self-assessment: Implementation and evaluation in an undergraduate nursing course. *Nurse Education Today*, *29*(6), 585-589. https://doi.org/10.1016/j.nedt.2008.12.008

# 21. Funding

This research received no specific external funding or commercial sponsorship for its execution. The study is conducted within the framework of the Teaching Innovation Project (PID 25-145) at the University of Granada, utilizing standard educational resources from the Faculty of Sport Sciences. JF-O is supported by the Spanish Ministry of Science, Innovation and Universities (FPU22/03052).

The authors declare there are not confilct of interest

# Appendix III. Training Log Sheet

The operational training log will be implemented in **Google Sheets**. The structure is illustrated below. Fields marked with “___” correspond to dropdown menus in which participants must select the practiced pattern.

**Fig. A3.** Daily juggling training log template (Google Sheets): date, session duration, and cumulative training time.

# Appendix IV. PANAS Emotion Questionnaire

**Table A5.** PANAS items (Positive Affect and Negative Affect) administered during the juggling evaluation (scale 1–5: NEVER–ALWAYS).

| **Nº** | **Item** | **Dimension** |
| --- | --- | --- |
| 1 | Interested | Positive Affect |
| 2 | Distressed | Negative Affect |
| 3 | Excited | Positive Affect |
| 4 | Upset | Negative Affect |
| 5 | Energetic | Positive Affect |
| 6 | Guilty | Negative Affect |
| 7 | Afraid | Negative Affect |
| 8 | Hostile | Negative Affect |
| 9 | Enthusiastic | Positive Affect |
| 10 | Proud | Positive Affect |
| 11 | Irritable | Negative Affect |
| 12 | Alert | Positive Affect |
| 13 | Ashamed | Negative Affect |
| 14 | Inspired | Positive Affect |
| 15 | Nervous | Negative Affect |
| 16 | Determined | Positive Affect |
| 17 | Attentive | Positive Affect |
| 18 | Jittery | Negative Affect |
| 19 | Active | Positive Affect |
| 20 | Fearful | Negative Affect |

# Appendix V. Flow Questionnaire

**Table A6.** Flow questionnaire items (adapted to juggling activity) administered after practice/evaluation (scale 1–5: STRONGLY DISAGREE–STRONGLY AGREE).

| **Nº** | **Ítem** |
| --- | --- |
| 1 | While juggling, my problems and worries disappear. |
| 2 | While juggling, I have a clear sense of how well or poorly I am performing. |
| 3 | While juggling, I act spontaneously and automatically, without having to think too much. |
| 4 | While juggling, I maintain total concentration. |
| 5 | While juggling, the way time passes feels different than usual. |
| 6 | While juggling, I feel sufficiently competent to meet the demands of the situation. |
| 7 | While juggling, the experience is extremely rewarding. |
| 8 | While juggling, I feel a complete sense of control. |
| 9 | While juggling, I have a clear sense of what I want to do. |
| 10 | While juggling, my thoughts are completely absorbed in the activity. |

# Appendix VI. Informed Consent for Research Participation

**Introductory Text**

This form records your decision on whether to authorize the use of data generated within the *Motor Games* course (University of Granada) for research purposes, as part of the TuJugling study (comparison of two juggling learning methods and the effect of learning order A→B vs. B→A). Participation in research is voluntary and has no academic consequences. Your decision here refers exclusively to authorizing the use of data for research purposes.

**What does the study involve?**

TuJugling is a study conducted within the *Motor Games* course (UGR) comparing two juggling practice methods:

- Intervention A: air-throw-based juggling
- Intervention B: ground-bounce-based juggling

The design is a class-group cross-over (A→B or B→A) with assessments at three time points: baseline, Evaluation 1, and Evaluation 2.

**What data would be used if you consent?**

If you consent, the following data may be used for research:

1. Juggling performance scores derived from evaluation videos
2. Questionnaires (PANAS and Brief Flow Inventory)
3. Exploratory measures (handgrip strength and a brief cognitive battery)

**Risks and discomfort**

Risk is low and comparable to usual juggling practice. It may include mild fatigue, minor ball impacts, and a risk of stumbling or loss of balance, particularly during BOSU tasks. You may stop at any time if you experience pain, dizziness, or discomfort.

**Voluntariness and withdrawal**

Participation is voluntary. You may withdraw consent at any time without academic consequences. If consent is withdrawn, your data will not be used in future analyses. Complete deletion may not be possible if data have already been anonymized or included in aggregated analyses.

**Confidentiality and video use**

Data will be processed in pseudonymized form using a study ID. The identity–ID correspondence table will be stored separately with restricted access. Videos will be used exclusively for performance scoring. Results will be reported in aggregated, non-identifiable form.

**Data protection**

- Controller: University of Granada
- Purpose: Scientific research and university teaching related to the TuJugling project
- Legal basis: Participant consent
- Recipients: Authorized research team
- Rights: Access, rectification, erasure, restriction, and withdrawal of consent

**Table A8**. Structure of the informed consent form (Google Forms)

| **Nº** | **Field** | **Purpose** |
| --- | --- | --- |
| 1 | Surname, Name | Identification for consent registration |
| 2 | ID/passport (optional) | Formal identification (only if necessary) |
| 3 | Contact email | Communication for queries/withdrawal |
| 4 | Confirmation of understanding | Evidence of comprehension and voluntariness |
| 5 | Consent decision | Primary consent variable |
| 6 | Signature date | Timestamp |
| 7 | Final declaration | Digital consent closure |

# Appendix VII. TuJugling Project Timeline

**Table A9.** Schedule of enrollment, intervention, and assessments.

| **Activity / Variable** | **Baseline (Week 0 2-5 march)** | **Period 1 (Week 1-4, 6 march-7 april)** | **Eval. 1 (Week 5, 8-14 april)** | **Wash-out (Week 6, 15-21 april)** | **Period 2 (Weeks 7-10, 22 april-19 may)** | **Eval. 2 (Weeks 11, 20-26 may)** |
| --- | --- | --- | --- | --- | --- | --- |
| Participant information & consent | X |  |  |  |  |  |
| Sequence allocation | X |  |  |  |  |  |
| Initial supervised session | X |  |  |  |  |  |
| Prescribed training |  | X |  |  | X |  |
| Juggling performance assessment | X |  | X |  |  | X |
| PANAS (post-assessment) | X |  | X |  |  | X |
| Flow questionnaire (post-assessment) | X |  | X |  |  | X |
| Handgrip strength | X |  |  |  |  | X |
| Cognitive battery | X |  |  |  |  | X |
| Adverse events log | X | X | X | X | X | X |
